# Analysis of Binding Determinants for Different Classes of Competitive and Noncompetitive Inhibitors of Glycine Transporters

**DOI:** 10.3390/ijms23148050

**Published:** 2022-07-21

**Authors:** Kamil Łątka, Marek Bajda

**Affiliations:** Department of Physicochemical Drug Analysis, Faculty of Pharmacy, Jagiellonian University Medical College, Medyczna 9, 30-688 Cracow, Poland; kamil.latka@doctoral.uj.edu.pl

**Keywords:** glycine transporters modeling, GlyT-1 inhibitors, GlyT-2 inhibitors, molecular docking, molecular dynamics

## Abstract

Glycine transporters are interesting therapeutic targets as they play significant roles in glycinergic and glutamatergic systems. The search for new selective inhibitors of particular types of glycine transporters (GlyT-1 and GlyT-2) with beneficial kinetics is hampered by limited knowledge about the spatial structure of these proteins. In this study, a pool of homology models of GlyT-1 and GlyT-2 in different conformational states was constructed using the crystal structures of related transporters from the SLC6 family and the recently revealed structure of GlyT-1 in the inward-open state, in order to investigate their binding sites. The binding mode of the known GlyT-1 and GlyT-2 inhibitors was determined using molecular docking studies, molecular dynamics simulations, and MM-GBSA free energy calculations. The results of this study indicate that two amino acids, Gly373 and Leu476 in GlyT-1 and the corresponding Ser479 and Thr582 in GlyT-2, are mainly responsible for the selective binding of ligands within the S1 site. Apart from these, one pocket of the S2 site, which lies between TM3 and TM10, may also be important. Moreover, selective binding of noncompetitive GlyT-1 inhibitors in the intracellular release pathway is affected by hydrophobic interactions with Ile399, Met382, and Leu158. These results can be useful in the rational design of new glycine transporter inhibitors with desired selectivity and properties in the future.

## 1. Introduction

Glycine is one of the major inhibitory neurotransmitters in the central nervous system (CNS). Once glycine is released from the presynaptic terminals, it binds to ionotropic glycine receptors, resulting in hyperpolarization of the postsynaptic membrane and neuronal inhibition [1]. Thus, glycine regulates the transmission of sensory and pain signals and ensures proper motor activity during movement [2]. In addition, it modulates the activity of the excitatory glutamatergic system by acting as a coagonist of the *N*-methyl-*D*-aspartate (NMDA) receptors [3] and consequently influences memory, learning, synaptic plasticity, and neuronal development [4].

The level of glycine in inhibitory and excitatory synapses is regulated by the glycine transporters of the sodium-dependent solute carrier family (SLC6). These transporters symport substrates against their concentration gradient based on the difference between the concentration of sodium and chloride ions on either side of the cell membrane [5]. There are two primary types of glycine transporters: GlyT-1 and GlyT-2 [2]. GlyT-2 transporters are found in the presynaptic terminals of glycinergic neurons located in the spinal cord, brainstem, and cerebellum, while GlyT-1 transporters are found mainly in membranes of glial cells surrounding the synapses of both glycinergic and glutamatergic systems. GlyT-1 transporters are also present near the NMDA receptors in the pre- and postsynaptic membranes of neurons [2]. Therefore, GlyT-1 transporters, in addition to their coincident localization with GlyT-2 transporters, can also be found in other regions of the CNS, such as the hippocampus, striatum, and prefrontal cortex [6].

As glycine transporters modulate the activity of both inhibitory and excitatory pathways, they have long been regarded as important therapeutic targets [6,7,8,9]. Considering the localization of GlyT-1 transporters in the vicinity of NMDA receptors, using the inhibitors of GlyT-1 transporters may be a new approach in the treatment of schizophrenia, which is i.a. characterized by impaired glutamatergic transmission [10]. The increased glycine level in the synaptic cleft after the administration of a GlyT-1 inhibitor leads to the saturation of coagonist binding sites at the NMDA receptors, thus facilitating their activation. In addition, modulating the NMDA receptor activity indirectly affects the mesolimbic dopaminergic transmission, which may be beneficial in the treatment of drug addiction, including alcoholism [9,10]. On the other hand, enhancement of glycinergic transmission by GlyT-1 inhibitors may aid in the treatment of epilepsy or neuropathic pain [6,7,9]. In the case of neuropathic pain, the approach of blocking GlyT-2 transporters seems to be of higher potential. Due to the restrictive localization of GlyT-2, which is largely limited to the structures responsible for nociception, i.e., the spinal cord and brainstem, GlyT-2 inhibitors appear to be safer and more advantageous [11]. It is worth noting that the complete impairment of GlyT-2 function is one of the causes of hyperekplexia—a serious hereditary neurological disease [12]. This disease is characterized by tremors, spasms, and episodes of apnea triggered by sudden and unexpected sensory stimuli. However, animal studies and subsequent clinical trials in humans have shown that a low therapeutic dose of GlyT-2 inhibitors is well tolerated and potentially alleviates various types of pain [7,11,13,14].

**Figure 1:**
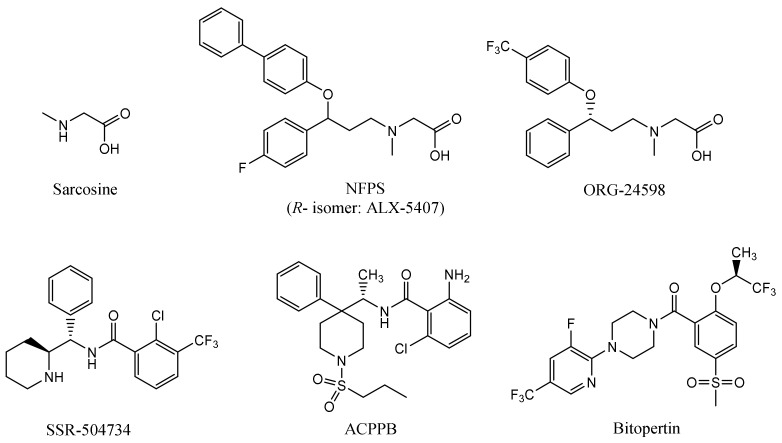
Examples of selective GlyT-1 inhibitors.

**Figure 2:**
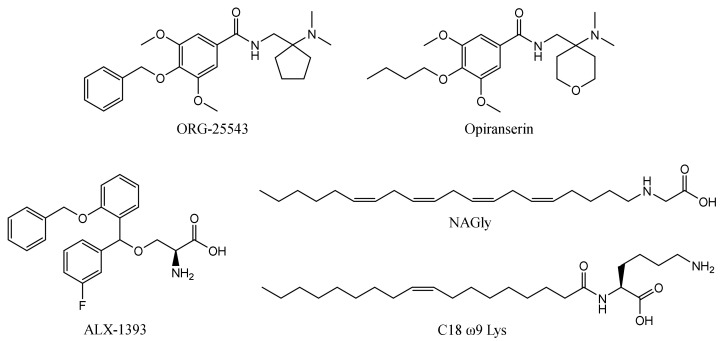
Examples of selective GlyT-2 inhibitors.

The first reported glycine transporter inhibitors were sarcosine and its derivatives with an attached aromatic fragment, such as ORG-24598, NFPS, or its *R*-isomer ALX-5407 (Figure 1) [6,8,15,16]. They were found to be selective GlyT-1 inhibitors. None of the sarcosine derivatives have been approved for therapeutic use, mainly due to their toxic effects which are manifested as respiratory depression, ataxia, and coma [17,18]. Interestingly, although sarcosine is a competitive inhibitor (substrate), its lipophilic derivatives are generally noncompetitive. In addition, many of these derivatives cause irreversible inhibition, which is indicated to be one of the causes of their toxic effects [6,16]. Therefore, several chemical groups of GlyT-1 inhibitors without the sarcosine fragment were synthesized and tested. These include aminophenethylbenzamide derivatives, such as SSR-504734, or other compounds containing a benzamide fragment with additionally substituted sulfonamide or sulfonyl groups, such as ACPPB (Figure 1) [6,8]. Among the best-investigated inhibitors with a nonsarcosine structure are the benzoylpiperazine derivatives, bitopertin and iclepertin [19,20,21]. They reached phase 3 clinical trials for the treatment of negative symptoms of schizophrenia. Bitopertin was finally found to have no significant therapeutic effect, while iclepertin is still on trial [22]. Compounds with a nonsarcosine structure, which include both competitive (most of the inhibitors) and noncompetitive (e.g., bitopertin and iclepertin) inhibitors, cause reversible inhibition [8,19,23,24,25,26,27,28]. Selective GlyT-2 inhibitors are limited in number and include compounds with an amino acid structure, of which the best known is ALX-1393, as well as those with a nonamino acid structure, such as ORG-25543 (Figure 2) [29,30,31]. ORG-25543 exhibits very high selectivity for GlyT-2; however, it blocks the transporter in an apparently irreversible manner, thus resulting in motor and respiratory side effects. Several ORG-25543 derivatives show more favorable reversible kinetics [13,32,33]. One among them is opiranserin, which is in phase 3 of clinical trials for the treatment of postoperative pain [14]. ALX-1393 is a reversible inhibitor, but exhibits a lower selectivity for GlyT-2 and poor permeability through the blood–brain barrier, which limits its clinical application [11,32]. Another interesting group of GlyT-2 inhibitors are derivatives with a lipid structure. The precursor of this group is *N*-arachidonyl glycine (NAGly), an endogenous lipid found mainly in the spinal cord, where it may regulate nociceptive pathways [34]. Modifications in the acyl tail and head group of the NAGly resulted in a series of highly active and selective derivatives, such as C18 ω9 L-Lys (Figure 2) [35,36,37].

**Figure 3:**
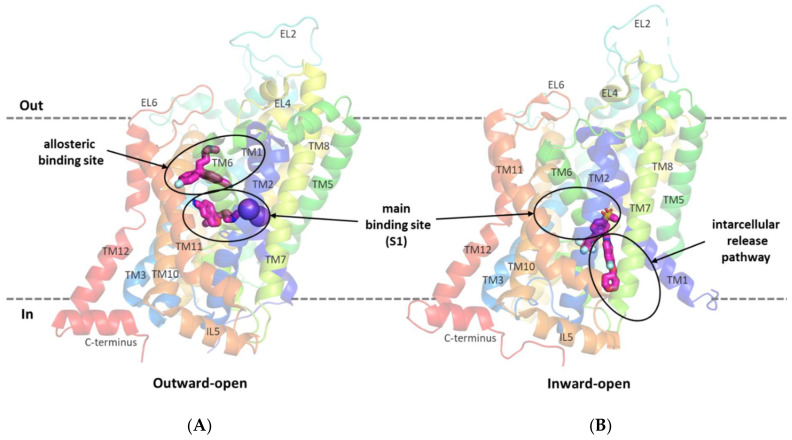
SERT in an outward-open state in complex with s-citalopram at the main and allosteric binding sites (PDB code: 5I73) (**A**). GlyT-1 transporter in an inward-open state in complex with a bitopertin analog (compound **1**) located partially within the intracellular glycine release pathway (PDB code: 6ZBV) (**B**).

The first relevant information about the structure of glycine transporters and the supposed binding mode of the substrate and inhibitors was obtained from the analysis of the crystal structures of related proteins from the SLC6 family, i.e., leucine transporter (LeuT), dopamine transporter (DAT), and serotonin transporter (SERT) [38,39,40,41]. All SLC6 transporters have 12 transmembrane domains with the *N*- and *C*-terminus located intracellularly. Other characteristic features of these transporters are the long extracellular loop EL2, which contains a glycosylation site, and the V-shaped extracellular loop EL4. TM1 and TM6 have nonhelical fragments (hinge regions) in about half of their length, which—along with the adjacent fragments of TM3, TM8, and TM10—form the main binding site (S1). In the vicinity of the S1 site, binding sites for sodium and chloride ions involved in substrate transport are also present [38,39,40,42,43]. For the transport of one glycine molecule, GlyT-1 requires two sodium ions and one chloride ion, whereas GlyT-2 needs three sodium ions and one chloride ion [44]. During transport, the transporter assumes the following conformational states: outward-open, allowing the ions and the substrate to bind from outside the cell; occluded; and inward-open, allowing the ions and the substrate to be released into the cell [5,42,45,46]. Crystal structure analysis and in vitro studies showed that inhibitors approach transporters from the extracellular side, binding at the main binding site or at the allosteric binding site within the vestibule (Figure 3A). Thus, most of the inhibitors block the transporters in an outward-open state [39,40,42,45]. An interesting exception is ibogaine, a noncompetitive inhibitor, which inhibits the SERT in its inward-open state, while remaining within the S1 site [47,48].

A breakthrough in the study of the structure of glycine transporters was the recent obtaining of the crystal structure of GlyT-1 in complex with an analog of bitopertin [49]. This crystal structure confirmed the overall compliance of the GlyT-1 structure with that of the previously explored SLC6 transporters and revealed an unusual binding mode of the inhibitor. The bitopertin analog (a noncompetitive inhibitor) blocks GlyT-1 in the inward-open state being located partially within the main binding site and partially at the intracellular site, which was not found before (Figure 3B) [49].

Despite these new findings, information on the binding modes of the inhibitors from other chemical groups and about the amino acids that determine the selectivity of glycine transporters is limited. Only a few articles based on mutagenesis data and molecular modeling studies have been published postulating the binding sites for ORG-25543, ALX-1393, and lipid-based GlyT-2 inhibitors [29,50,51]. There is a lack of studies that extensively compare the structure of the two types of glycine transporters, taking into account both recent experimental data and a larger ligand library.

Therefore, to investigate the exact structure of the binding sites of GlyT-1 and GlyT-2, we decided to build a pool of homology models of both transporters using the recently released structure of GlyT-1 in the inward-open state and the crystal structures of DAT and SERT in other conformational states. Then, using docking studies and molecular dynamics (MD) simulations, we identified the binding modes of different groups of inhibitors, as well as the amino acids that determine the selectivity of glycine transporters.

## 2. Results and Discussion

### 2.1. Model Building

To build the models of GlyT-1 and GlyT-2 in the outward-open state, we used three crystal structures of DAT (PDB codes: 4M48, 4XP4, and 4XP9) and one structure of SERT (PDB code: 5I73) as templates. In addition, a model in the partially occluded state was also created based on DAT (PDB code: 4XPH). We used several templates to increase the diversity of our pool of models to achieve a higher possibility of finding the structure most similar to the real one. LeuT was rejected as a template due to its significantly lower amino acid sequence homology with glycine transporters. The models were built using Modeller program and the SWISS-MODEL server and were then evaluated by tools checking their quality and similarity to real protein structures. The best GlyT-1 and GlyT-2 models were chosen by docking a pool of selective ligands. The following criteria were considered for choosing the best models: The consistency of poses obtained for structurally related compounds, their docking scores, and the ability to explain structure–activity relationships. For competitive inhibitors, substrate-specific interactions with sodium ions and Gly121 were also taken into account. The best GlyT-1 model turned out to be the one built on the 4M48 template, and the best GlyT-2 model was the one built on the 4XP9 template, both with SWISS-MODEL server. Models of GlyT-2 in the inward-open state were built on both available GlyT-1 templates (PDB codes: 6ZBV and 6ZPL). Ligand docking in accordance with the aforementioned criteria revealed that the best model was the one built on the 6ZPL template using the SWISS-MODEL server. The crystal structures of GlyT-1 in the inward-open state possess a rather low resolution (3.40 and 3.94 Å) and lack a fragment of the long loop EL2, as well as the amino acids forming the intracellular loop between TM4 and TM5. Moreover, they have four-point mutations in the amino acid sequence. To fill in/replace the missing fragments and to slightly optimize the residue side chains, we decided to build a complete model of GlyT-1 in the inward-open state based on the 6ZBV and 6ZPL crystal structures. For detailed analyses, we used the model built on the 6ZPL template with the SWISS-MODEL server, which proved to be the most universal one for redocking of the compound from the crystal structures, as well as the analogs of this compound.

### 2.2. Structure of GlyT-1 and GlyT-2 Binding Sites

As the general structure of glycine transporters is well known, we focused on analyzing the structure of the binding sites: the main binding site (S1); the transporter vestibule including the S2 site; and the binding site overlapping with the intracellular release pathway.

**Figure 4:**
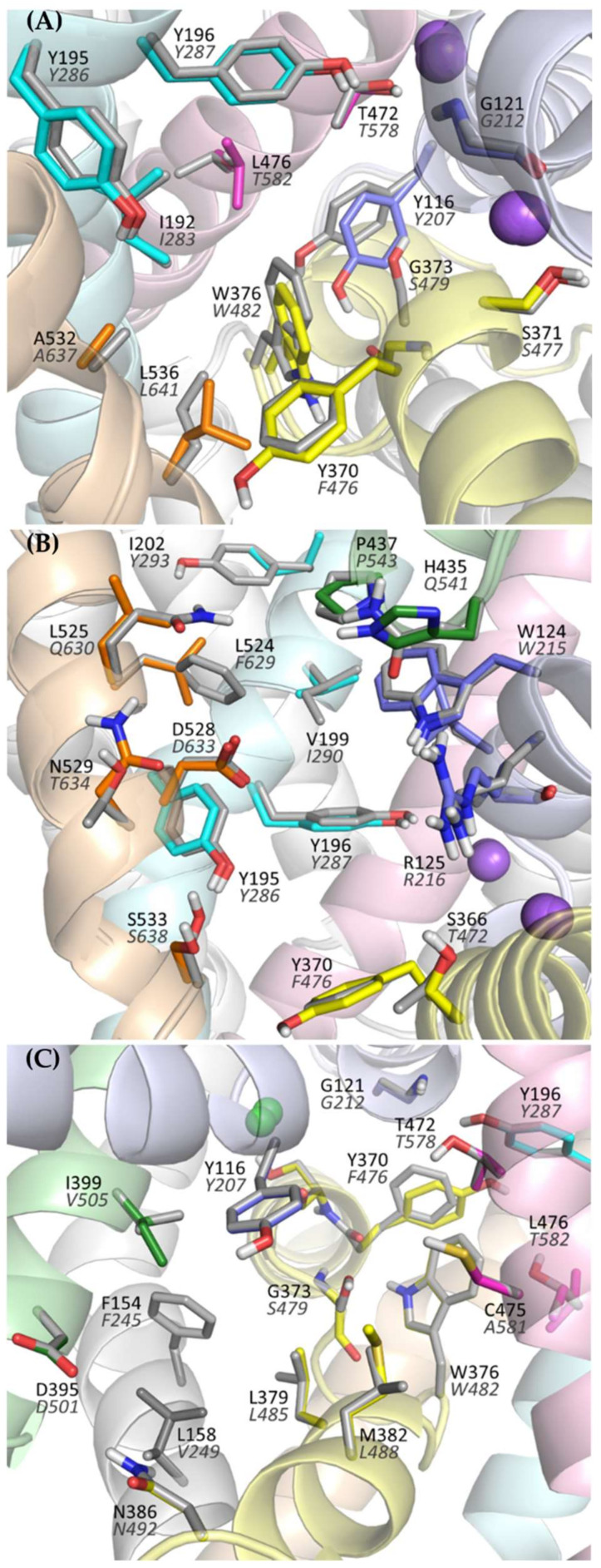
Amino acids forming the main binding site (S1) (**A**), vestibule (**B**), and intracellular release pathway (**C**) of the glycine transporters. For GlyT-1, residues are presented in color (according to transmembrane domains), whereas for GlyT-2 gray is used for clarity. Labels for GlyT-2 are shown in gray italics.

Analyzing the structure of the main binding site in glycine transporters, it can be seen that the volume of the binding site is significantly reduced compared with transporters for dopamine, serotonin, or even leucine and gamma-aminobutyric acid (GABA). This is mainly due to the presence of a tryptophan residue (Trp376 in GlyT-1 and Trp482 in GlyT-2) in place of the phenylalanine residue in DAT, SERT, and LeuT or leucine/glutamine in GABA transporters. To a lesser extent, the volume of S1 is also reduced by the presence of threonine (Thr472 in GlyT-1 and Thr578 in GlyT-2) in place of serine in the other transporters (Figure 4A). This tighter binding site is clearly an adaptation for the selective transport of small glycine molecules. Mutagenesis studies indicate that the replacement of Trp482 in GlyT-2 with phenylalanine decreases the affinity for glycine itself while extending its ability to transport other amino acids, including alanine, leucine, methionine, and even phenylalanine [52]. The replacement of Thr578 with serine in GlyT-2 reduced its selectivity to glycine, thus extending the possibility of transport of other amino acids, although their affinity remained low (EC_50_ > 1 mM). An important difference at the S1 site between GlyTs and monoamine transporters is the presence of Leu476 in GlyT-1 or Thr582 in GlyT-2, at the position of glycine in DAT and SERT. This leads to a significant reduction in the volume of one of the pockets within the S1 site where the aromatic fragments of DAT and SERT inhibitors bind [39,40,42]. Therefore, it is unlikely that similar fragments of inhibitors could be accommodated in this pocket in GlyTs. A similar reduction in this pocket, followed by the obstruction of ligand binding therein, is observed in other transporters for aliphatic amino acids, such as LeuT (Ile359), and GABA transporters (Thr400 in GAT-1, Cys399 in BGT-1, Cys394 in GAT-2, and Cys414 in GAT-3) [38,45,53,54]. Interestingly, the replacement of Thr582 with leucine in GlyT-2 leads to substantial or even complete impairment of glycine transport [29,52]. It is worth mentioning that GlyT-2, in contrast to GlyT-1, is much more selective for glycine [52]. Besides glycine, GlyT-1 can transport sarcosine (*N*-methylglycine) and *N*-ethylglycine. A major determinant of this selectivity is the presence of Ser479 in GlyT-2 at the position of Gly373 in GlyT-1. Ser479 further limits the volume of the S1 site in GlyT-2, creating a steric clash for the methyl/ethyl fragment attached to the amino group of glycine. Mutagenesis studies indicate that the replacement of Ser479 with glycine reduces several times the affinity of GlyT-2 for glycine, but restores its ability to transport sarcosine and, to a lesser extent, *N*-ethylglycine [52,55]. A reverse mutation in GlyT-1 (Gly373Ser) leads to a complete blockade of the transporter. Another difference in the S1 site between GlyT-1 and GlyT-2 concerns the amino acid that forms the extracellular gate: Tyr370 in GlyT-1 versus Phe476 in GlyT-2. According to mutagenesis studies, the replacement of tyrosine with phenylalanine does not affect the function of GlyT-1 and even increases its affinity for glycine. Interestingly, the reverse mutation in GlyT-2 (Phe476Tyr) results in significant impairment or even a complete blockade of the transporter function [29,55].

Significant structural differences between GlyT-1 and GlyT-2 are found at the S2 site, which is located above the extracellular gate, within the vestibule of the transporter. The volume of this site is much larger in GlyT-1. This is mainly due to the presence of Leu524 and Val199 in GlyT-1 in place of Phe629 and Ile290 in GlyT-2, respectively (Figure 4B). In addition, in GlyT-1, above these residues, there is an aliphatic residue Ile202, which is replaced in GlyT-2 by a larger aromatic residue Tyr293, which also reduces the volume of the S2 site in GlyT-2. These differences, as confirmed by the docking results described further, have a substantial impact on the selective binding of ligands.

A recently released crystal structure of GlyT-1 in the inward-open state revealed the possibility of binding inhibitors within the intracellular release pathway. This new site partially covers the main S1 binding site. Therefore, the presence of Ser479 in GlyT-2 in place of Gly373 in GlyT-1 at the S1 site, affects ligand binding also in the inward-open state. Comparing the structure of both transporters in the area closer to the intracellular side, it can be observed that this region is tighter in GlyT-1 due to the side chains of Ile399, Met382, and Leu158, which correspond to Val505, Leu488, and Val249, respectively, in GlyT-2 (Figure 4C). This enables GlyT-1 to create stronger hydrophobic interactions with ligands.

### 2.3. Ligand Binding Studies

A selected pool of inhibitors of each type of GlyTs was docked to the abovementioned binding sites of transporters in both outward- and inward-open states. For the chosen compound representatives, MD simulations were performed to check the stability of the complexes in each state. In addition, binding free energies were compared based on the MM-GBSA calculations for the last 2 ns of the MD simulations. The obtained results corresponded well with structure–activity relationships and kinetics data for particular groups of glycine inhibitors. Structures of the compounds for which the detailed modes of binding with glycine transporters are described herein are shown in Supplementary Figure S2.

### 2.4. Binding Mode of Noncompetitive GlyT-1 Inhibitors

**Figure 5:**
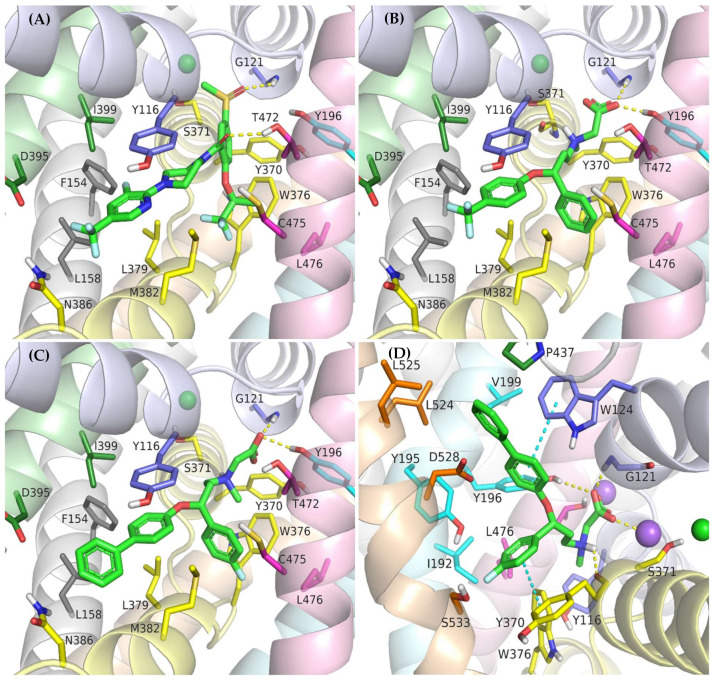
Binding mode of the noncompetitive GlyT-1 inhibitors within the transporter in the inward-open state: bitopertin (**A**), ORG-24598 (**B**), ALX-5407 (**C**); and transporter in the outward-open state: ALX-5407 (**D**).

**Figure 6:**
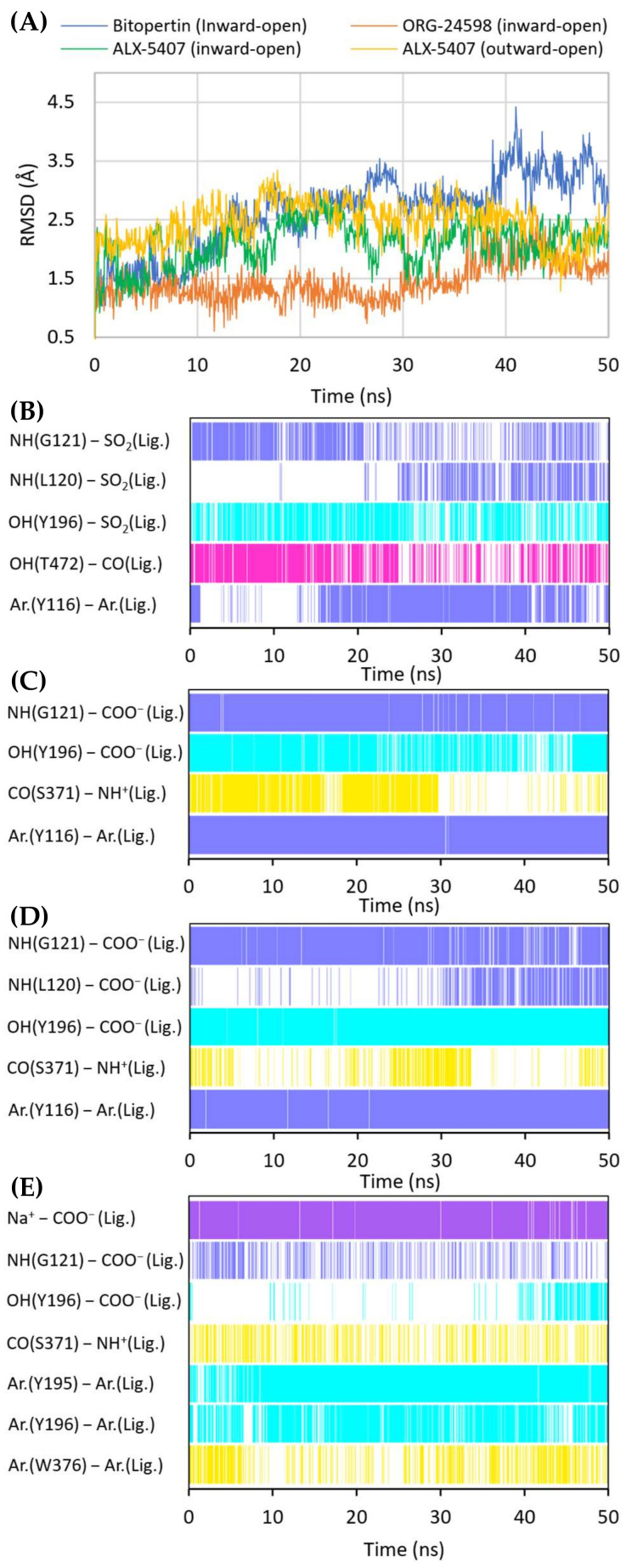
RMSD changes observed for the ligands in a complex with GlyT-1 in the course of MD simulations (**A**). Stability of the key interactions between the ligand and the transporter during MD simulations for bitopertin (**B**), ORG-24598 (**C**), and ALX-5407 (**D**) in a complex with GlyT-1 in the inward-open state and for ALX-5407 in a complex with GlyT-1 in the outward-open state (**E**). The presence of interactions in a particular MD frame is marked in color.

The crystal structures of GlyT-1 in the inward-open state represent the binding mode of compound **1**, which is a noncompetitive inhibitor with a benzoylisoindoline scaffold [49]. After docking other derivatives with the same chemotype, including bitopertin, a binding mode highly consistent with that observed in the crystal structures was obtained (Figure 5A, Supplementary Figure S3). The methylsulfonyl group of the compounds forms a hydrogen bond with Gly121 from the nonhelical fragment of TM1 within the S1 site. In the MD simulations carried out for compound **1** and bitopertin, hydrogen bonds with adjacent Leu120 as well as the hydroxyl group of Tyr196 from TM3 were observed (Figure 6B). It is worth noting that this pocket is tight enough to prevent the binding of derivatives that contain larger than methyl substituents. The position of the sulfonyl group and its interactions correspond to those of the carboxylic group of leucine or tryptophan in the LeuT crystal structures, as well as the predicted binding mode of the carboxylic group of nipecotic acid derivatives in GABA transporters [38,45,53]. However, it should be noted that tryptophan or GABA inhibitors bind to the transporters from the extracellular side, thus blocking them in the outward-open state. The alkyl or cycloalkyl fragments of the compounds, often substituted with fluorine atoms, as well as other cyclic moieties such as morpholine, are located between TM6 and TM8 and form hydrophobic interactions mainly with Trp376, Leu379, Leu476, and Cys475. The benzoyl fragment creates hydrophobic interactions with Trp376, and in the case of some derivatives, also CH–π stacking with this residue. It is also worth pointing out that within the nonhelical fragment of TM6, there is Gly373. As described earlier, this amino acid in GlyT-2 is replaced with a serine residue (Ser479), which may create a steric clash and significantly obstruct the binding of the compounds in GlyT-2, thus affecting their selectivity. The remaining component of the inhibitors, corresponding to 3-fluoro-5-(trifluoromethyl)pyridine-2-yl)piperazine fragment in bitopertin, is directed toward the inside of the cell. This fragment fits into the hydrophobic pocket formed by Tyr116 in TM1, Leu379 and Met382 in TM6, Leu158 and Phe154 in TM2, and Ile399 in TM7. Additionally, the aromatic ring can create π–π or CH–π stacking with Tyr116. As mentioned earlier, this pocket is tighter in GlyT-1 compared with GlyT-2, which enables better fitting of the inhibitors and, together with the Gly373 and Ser479 substitution, is responsible for their selective binding. In the MD simulation, the position of compound **1** was very stable (Supplementary Figure S5). Bitopertin showed a higher RMSD change; however, this was mainly due to the rotation of the aromatic ring (Figure 6A). The most relevant interactions were preserved (Figure 6B).

When bitopertin analogs were docked to the S1/S2 site of the GlyT-1 models in the outward-open state, less consistent binding modes were obtained. Some compounds, such as compound **1** and bitopertin, interact with Gly121 and the sodium ion via a sulfonyl group (Supplementary Figure S4). The fluorinated alkyl substituents at position 2 of the benzoyl fragment were located at the level of the extracellular gate (Tyr196–Tyr370 line), being directed toward TM10 or within the S2 site reaching Trp124. The remaining fragment of the compounds was located above Tyr370 and, in some cases, it creates π–π stacking with this residue. In the MD simulation, compound **1** did not retain its initial position (Supplementary Figure S5). The interactions within the S1 site were broken, and the compound moved closer to the vestibule entrance. For bitopertin, the position, as well as the interactions, was preserved during the MD simulation. However, the MM-GBSA energy value of both compounds was significantly lower (more beneficial) for complexes in the inward-open state, which was particularly true in the case of compound **1** (Table 1). This may confirm the preferential binding of bitopertin analogs within the intracellular release pathway.

Noncompetitive GlyT-1 inhibitors also include sarcosine derivatives. While docking to the GlyT-1 structure in the inward-open state, these inhibitors adopt an arrangement similar to that of the analogs of bitopertin (Figure 5B,C). The carboxyl group is located in the position of the sulfonyl moiety, forming hydrogen bonds with Gly121 and Tyr196, as well as with Leu120 in some cases. In the MD simulations for ORG-24598 and ALX-5407, the protonated amino group of these compounds can create a hydrogen bond with the main chain of Ser371 (Figure 6C,D). Their aromatic rings occupy the same areas as the corresponding fragments of bitopertin analogs. The most significant difference is the lack of a hydrogen bond with Thr472. Kinetics studies indicate that although ORG-24598 inhibits glycine uptake in a noncompetitive manner, it blocks the binding of bitopertin in a competitive way [19]. Thus, the finding that the binding mode of ORG-24598 and its analogs is highly consistent with that of bitopertin seems reasonable. While docking to the GlyT-1 model in the outward-open state, sarcosine derivatives also adopted a consistent arrangement (Figure 5D, Supplementary Figure S4D). The carboxyl group coordinates the sodium ion and forms a hydrogen bond with Gly121. A hydrogen bond with the hydroxyl group of Tyr196 can also be observed, although it was significantly less stable in MD simulations (Figure 6E). The protonated amino group creates a hydrogen bond with the main chain of Ser371 (as in the inward-open state), being arranged similarly to the amino moiety of leucine in the crystal structures of LeuT [38]. One of the aromatic rings is positioned at the level of the extracellular gate forming hydrophobic interactions mainly with Trp376, Tyr116, and Ile192. For some derivatives, it additionally creates CH–π stacking with Trp376 (Figure 6E). The second aromatic fragment is located within the S2 site, where it forms numerous hydrophobic interactions with Val199, Leu524, Trp124, Tyr195, and Tyr196, as well as π–π and/or CH–π stacking with Trp124 or Tyr195 and Tyr196 residues (Figure 5D and Figure 6E). The volume of the pocket in which this aromatic fragment binds is significantly reduced in GlyT-2 mainly by the side chains of Ile290 (Val199 in GlyT-1) and Phe629 (Leu524 in GlyT-1). The RMSD for ORG-24598 located in S1/S2 is more variable than that for the same compound bound within the intracellular release pathway (Supplementary Figure S5). Furthermore, the MM-GBSA energy value is significantly lower for the complex in the inward-open state, indicating that this transporter state and binding site are preferred for ORG-24598 (Table 1). Different results were obtained for compound ALX-5407 (*R* isomer of NFPS). RMSD plots as well as MM-GBSA energy values show that this compound binds equally well in the outward-open and inward-open states (Table 1, Supplementary Figure S5). Some studies suggest that the binding of NFPS to GlyT-1 is independent of the presence of sodium ions, which may be in disagreement with the presented binding mode within the S1/S2 site [56,57]. At the same time, other studies indicate that TM1 and TM3 contain determinants that affect the affinity of NFPS to GlyT-1 [56]. TM3 contributes to S1 and S2 sites, while it is distant from the intracellular release pathway. Interestingly, replacing TM3 in GlyT-2 with that in GlyT-1 increases the affinity of NFPS to that of wild-type GlyT-1. Moreover, NFPS exhibits a mixed mode of inhibition against this chimera [56]. It is likely that the effect of NFPS on this chimera is related to the ability of the inhibitor to bind within the S1/S2 site since, together with the replacement of the entire TM3 in GlyT-2, Ile290 is substituted by Val, which increases the volume of the S2 site, thus allowing the accommodation of NFPS biphenyl fragment. Based on the results obtained, it can be concluded that NFPS/ALX-5407 probably has the ability to bind to GlyT-1 in both inward-open and outward-open states.

Many studies have reported the apparently irreversible modes of inhibition of sarcosine derivatives [15,16,57]. As these compounds do not contain chemical moieties that can form covalent bonds with proteins, other factors must be responsible for this phenomenon. The results of docking studies and MD simulations that these compounds bind from the intracellular side, together with data showing their ability to interfere with the cell membrane [57], may partially explain the difficulty in washing them out. However, this issue requires further biological study, as well as in silico analysis.

### 2.5. Binding Mode of Competitive GlyT-1 Inhibitors

**Figure 7:**
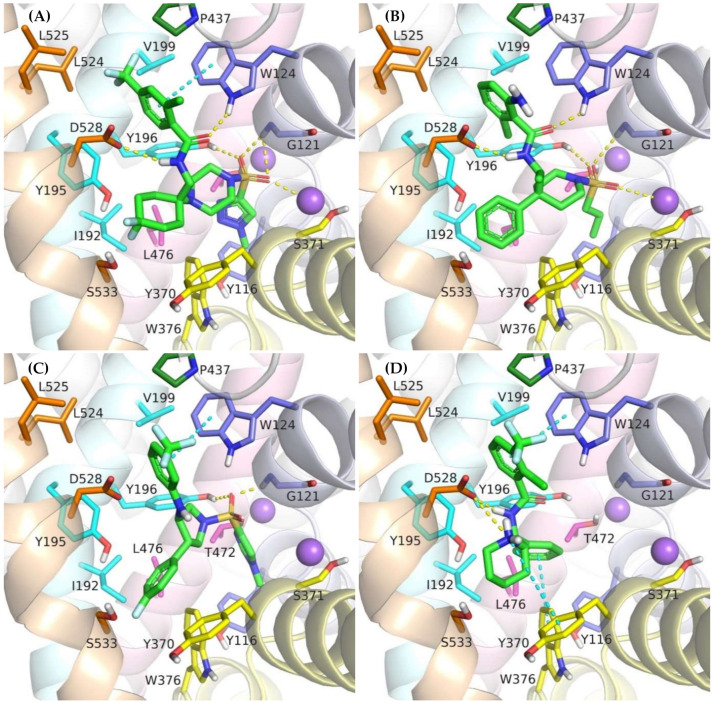
Binding mode of the competitive GlyT-1 inhibitors within the transporter in the outward-open state: compound **2** (**A**); ACPPB (**B**); compound **3** (**C**); SSR-504734 (**D**).

**Figure 8:**
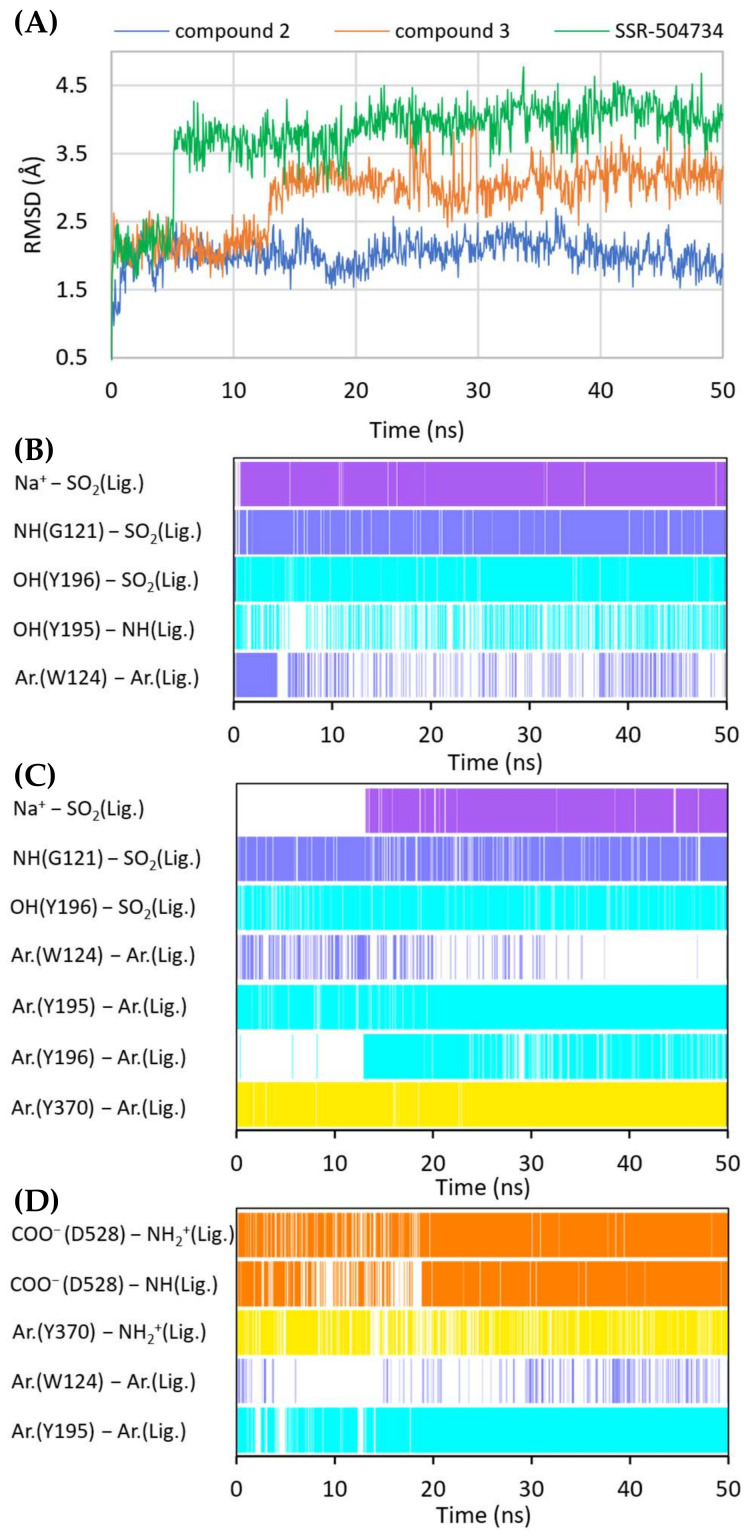
RMSD changes observed for the ligands in a complex with GlyT-1 in an outward-open state in the course of MD simulations (**A**). Stability of the key interactions between the ligand and the transporter for compound **2** (**B**), compound **3** (**C**), and SSR-504734 (**D**). The presence of interactions in a particular MD frame is marked in color.

Molecular docking and subsequent MD simulations revealed consistent binding modes for competitive GlyT-1 inhibitors in a model of this transporter in the outward-open state (Figure 7 and Figure 8, Supplementary Figure S6). The inhibitors occupy the S1 and the S2 sites partially. For derivatives containing a sulfonamide or sulfonyl group, this fragment binds near the nonhelical fragment of TM1, creating a hydrogen bond with Gly121 and coordinating the sodium ion. It also forms a hydrogen bond with the hydroxyl group of Tyr196 from TM3. This arrangement is similar to that of the sulfonyl group present in bitopertin and its analogs. Alkyl (propyl, cyclopropylmethyl) or heteroaromatic (*N*-methyltriazole, *N*-methylimidazole) substituents attached to the sulfonamide/sulfonyl group are located deeper in the S1 site, occupying the hydrophobic pocket constituted mainly by Tyr116, Trp376, and Leu476. In GlyT-2, binding of this fragment may be hindered by the side chain of Ser479, which is in place of Gly373. A decrease in the hydrophobicity of this pocket may also affect the binding of these fragments in GlyT-2, which could be due to the presence of Thr582 in place of Leu476 in GlyT-1.

A large number of the discussed sulfonamide derivatives have two hydrophobic/aromatic fragments linked to the sulfonamide or sulfonyl group through saturated heterocyclic (piperidine, piperazine, pyrrolidine), bicyclic, or cyclohexane moieties. One of these fragments is usually a benzamide substituted with halogen atoms or other similar aromatic moieties. This fragment is located within the S2 site, where it forms numerous hydrophobic interactions with Trp124, Tyr195, and Tyr196, and with Val199 and Leu524, which are crucial for selectivity (Figure 7). In addition, π–π and/or CH–π stacking interactions involving either Trp124 residue (compound **2**) or Tyr195 and Tyr196 residues (compound **3**) can be observed (Figure 8B,C). Docking results showed the presence of hydrogen bonds between the amide group of the compounds and the side chains of Asp528 and Trp124; however, they were not found to be stable in MD simulations. However, compound **2** forms another fairly stable hydrogen bond with the hydroxyl group of Tyr195, which is located close to the carboxyl moiety of Asp528 (Figure 8B). The second hydrophobic fragment of the discussed compounds, which is usually a cycloalkyl or phenyl ring, is directed toward TM10, which lies at the level of the extracellular gate (Tyr196–Tyr370) or slightly above it. It creates hydrophobic interactions mainly with Tyr370, Trp376, Ile192, and Leu532, and, in the case of compound **2**, with Tyr195. Compound **3** additionally forms π–π interactions with Tyr370 (Figure 8C).

Another group of competitive GlyT-1 inhibitors containing a sulfonamide moiety is the derivatives of tetrahydroquinoline, aminotetraline, and aminochromane, and their analogs. The difference between these compounds and the ones discussed earlier is the presence of a protonated amine group at a physiological pH. This group creates a salt bridge with the carboxyl moiety of Asp528 from the extracellular gate (Supplementary Figure S6B). Aromatic rings are involved in hydrophobic interactions with Tyr370, Trp376, and Ile192 and, to a lesser extent, with Trp124.

SB-733993 is a selective competitive GlyT-1 inhibitor that contains a naphthalene moiety attached directly to the sulfonamide group. This aromatic fragment forms hydrophobic interactions with Tyr116, Tyr196, and Trp376, and additionally CH–π stacking with Trp376 (Supplementary Figure S6D). The sulfonamide group is located close to the sodium ion and Gly121. The protonated piperidine, in turn, creates a salt bridge with Asp528. This residue is also involved in hydrogen bonds with the hydroxyl group of the compound. The 2,5-dimethylpiperidine fragment is positioned within the same area as the benzamide or phenyl rings of the previously discussed compounds.

A large subset of competitive GlyT-1 inhibitors are aminophenethylbenzamide derivatives, such as SSR-504734. Their benzamide fragment is located in the hydrophobic pocket of the S2 site (Figure 7D). MD simulation for SSR-50473 showed that this fragment creates CH–π interactions with Trp124 and Tyr195 (Figure 8D). The amide nitrogen atom forms a strong and stable hydrogen bond with Asp528, whereas the protonated amine group is involved in a stable salt bridge with this residue. In addition, the piperidine ring can form cation–π and hydrophobic interactions with Tyr370. The phenyl ring is oriented toward the S1 site, creating hydrophobic interactions with Trp376, Tyr196, and Leu476. Docking studies also indicated CH–π stacking with Trp376, but it was not stable in MD simulations due to a slight shift in the phenyl fragment toward Tyr370 from TM6. These results suggest that the discussed derivatives, despite being competitive inhibitors, mainly bind to the S2 site, forming only hydrophobic interactions with some amino acids from the S1 site or the extracellular gate. Typical interactions with the sodium ion or Gly121 could not be observed. However, there are several arguments defending the demonstrated binding mode of aminophenethylbenzamide derivatives. The first argument stems from in vitro studies which have shown that SSR-504734 analogs inhibit GlyT-1 regardless of the presence of sodium ions, in contrast to sulfonamide-containing inhibitors (GSK931145 and ACPPB) that require these ions for their activity [8,23,58]. Another argument is that the arrangement of benzamide and the piperidine rings is consistent with the arrangement of the corresponding fragments of the sulfonamide/sulfonyl derivatives (Figure 7). Moreover, one of the aminophenethylbenzamide derivatives contains the attached sulfonyl group substituted with cyclopropylmethyl at position 4 of the phenyl ring. In docking studies, the fragment of the parent structure retained the arrangement and interactions characteristic of all derivatives, whereas the attached alkylsulfonyl moiety reached the sodium ion and Gly121, forming interactions within the S1 site (Supplementary Figure S6E). The same was observed for derivatives with an additional aromatic ring at position 3 of the phenyl ring, usually pyrazole or imidazole (including the substituted one with alkyl groups). An extra aromatic fragment extends toward Tyr116, intensifying hydrophobic interactions, and the free electron pair of the nitrogen atom from the ring coordinates the sodium ion (Supplementary Figure S6F). A parallel observation is the binding of the tetrahydroquinoline derivatives. Interestingly, the parent compound for this group was a hit (tetrahydroisoquinolin-7-ol derivative) that lacks the sulfonamide fragment [27]. However, according to the pose obtained in docking studies, this compound binds similarly to its sulfonamide derivative (Supplementary Figure S6C). It exhibits a competitive mode of inhibition, but a week interaction with S1, as observed in the case of SSR-504734 analogs.

While docking structurally similar compounds to the GlyT-1 model in the inward-open state, we obtained significantly less consistent poses (Supplementary Figure S7). For some of the sulfonamide derivatives, the aromatic fragments overlapped with the arrangement of the aromatic fragments of compound **1** from the crystal structures of GlyT-1, but the sulfonamide group did not form analogous interactions within the S1 site. Other compounds adopted a different position, which indicates a mismatch with the binding mode observed for bitopertin analogs. This is primarily due to the larger alkyl/aromatic substituent at the sulfonamide/sulfonyl group found in competitive inhibitors, which cannot fit into a tight pocket within the nonhelical fragment of TM1. For SSR-504734, the aromatic rings generally overlapped with the aromatic fragments of compound **1**, but in the case of its analogs, the poses widely varied. Even though in the MD simulation the tested compounds generally remained in the binding site and reached stability in the second part of the simulation, their RMSD plots were more variable compared with the those obtained for complexes in the outward-open state (Supplementary Figure S8). Additionally, for all three tested representatives of the competitive inhibitors, the MM-GBSA energy values were significantly lower for the arrangement within the S1/S2 site in the outward-open state, which suggests that it is preferred (Table 1).

### 2.6. Binding Mode of GlyT-2 Inhibitors

**Figure 9:**
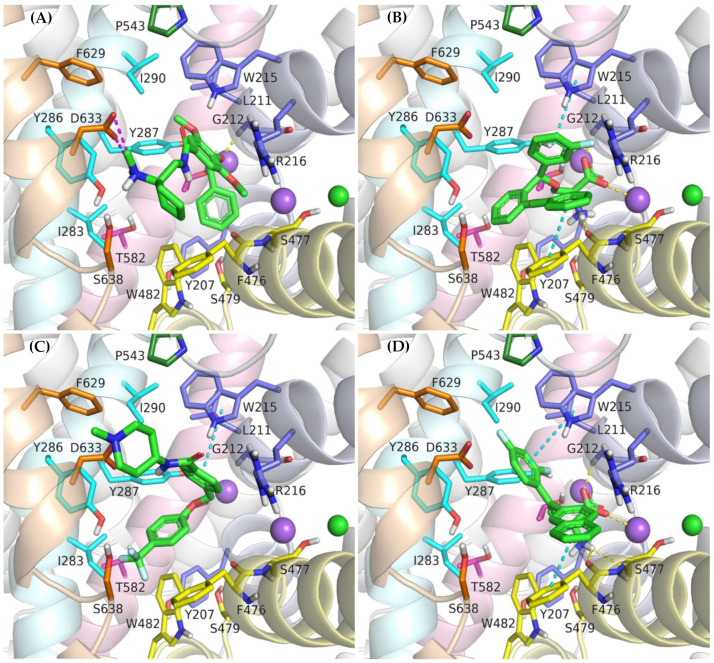
Binding mode of the inhibitors within GlyT-2 in an outward-open state: ORG-25543 (**A**); ALX-1393 (**B**); GT-0198 (**C**); compound **9** (**D**).

**Figure 10:**
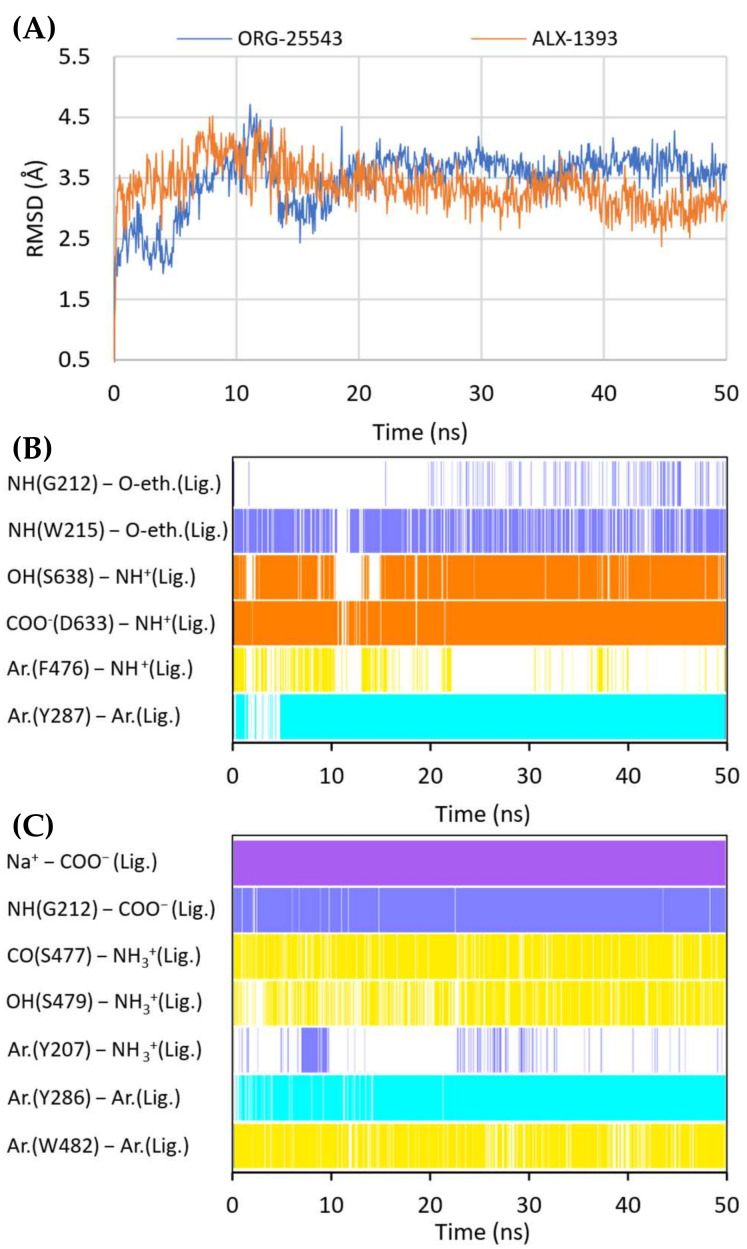
RMSD changes observed for ORG-25543 and ALX-1393 in a complex with GlyT-2 in the outward-open state in the course of MD simulations (**A**). Stability of the key interactions between the ligand and the transporter for ORG-25543 (**B**) and ALX-1393 (**C**). The presence of interactions in a particular MD frame is marked in color.

In vitro studies indicate that compound ORG-25543 and its analogs are highly selective and noncompetitive inhibitors of GlyT-2. Additionally, this compound behaves as an irreversible inhibitor in many assays. However, its derivatives, which have only a slightly modified structure, are fully reversible inhibitors [29,32,33]. In the docking of ORG-25543 and its derivatives to the GlyT-2 model in the outward-open state, the compounds were mainly arranged within the S2 site (Figure 9A and Figure 10). The binding mode was very similar to that proposed by Benito-Munoz et al. [29]. The protonated amine group creates an ionic interaction with Asp633 and a hydrogen bond with the hydroxyl group of Ser638. It is worth mentioning that the Asp633Glu mutation almost completely abolished the activity of ORG-25543, whereas Ser638Cys caused a significant impairment [29]. A possible cation–π interaction with Phe476 was also observed for the protonated amine group, although it was not very stable in the MD simulation (Figure 10B). The cyclopentane ring forms hydrophobic interactions with Phe476 and, as indicated by MD simulations, also with the side chain of Thr472. In GlyT-1, Thr472 residue is replaced by a serine residue, which weakens the possibility of hydrophobic interactions. One of the methoxy moieties attached to the benzamide fragment creates a hydrogen bond with the nitrogen atom (NH) of the Trp215 side chain (Figure 10B). Additionally, the entire benzamide fragment forms hydrophobic interactions with this residue. As expected, the Trp215Phe mutant showed a reduced affinity for this compound [29]. The second methoxy group and the oxygen atom of the 4-benzyloxy fragment are located near the nonhelical fragment of domain 1; however, they do not interact with the sodium ion, and the hydrogen bond with Gly121 was found to be relatively unstable during MD simulations (Figure 10B). This is in agreement with findings indicating that ORG-25543 binds to GlyT-2 regardless of the presence of sodium ions [29]. The benzyloxy fragment initially points toward Tyr207 but, during MD simulations, it moved slightly toward Tyr287, forming π–π stacking with this residue. The nearby Thr582 residue may play a key role in the selective binding of this fragment to GlyT-2, as observed in biological assays [29]. Replacement of this residue with leucine, found at this position in GlyT-1, impaired the binding of ORG-25543, probably due to the reduced volume of the cavity where the aromatic ring of the benzyloxy fragment is located. The structurally related compound GT-0198 shows an arrangement similar to that of ORG-2543 and its analogs (Figure 9C). The protonated amine of the piperidine ring forms a salt bridge with Asp633, whereas the benzyl fragment creates hydrophobic and CH–π interactions with Trp215. The second aromatic fragment reaches the S1 site, interacting mainly with Trp482, Ile283, and Tyr287. While docking to the GlyT-2 model in the inward-open state, the aromatic fragments of ORG-25543 and its analogs were arranged similar to one of the aromatic fragments of compound **1** in the crystal structure of GlyT-1 (Supplementary Figure S9). However, unlike in GlyT-1, one of the hydrophobic pockets between TM6 and TM8 was empty. In addition, the cycloalkyl ring of the compounds was located at a position corresponding to that of the polar sulfonyl moiety in GlyT-1. The only beneficial interaction observed at this site was the hydrogen bond between the protonated amine group and the main chain of Ser477. However, this bond, as well as the position of the entire ligand, was unstable during MD simulation (Supplementary Figure S10**)**. Compound GT-0198 did not seem to fit into the described binding site, extending toward the inside of the cell. Moreover, the MM-GBSA energy value was higher for the ORG-25543–GlyT-2 complex in the inward-open state than that calculated for the complex in the outward-open state (Table 2). All these observations indicate that the described derivatives approach GlyT-2 from the extracellular side and bind to it in the outward-open state.

Compound ALX-1393, as well as other related glycine derivatives, binds consistently within the S1 and S2 sites (Figure 9B,D). The carboxyl group exhibits characteristic interactions with the sodium ion and Gly212. Kinetics data suggest that although ALX-1393 is generally a noncompetitive inhibitor, at high concentrations it could compete with glycine. In addition, the sodium ion binding to GlyT-2 affects the affinity of ALX-1393 for this transporter [29]. Thus, the above-described interactions seem to be reasonable. The protonated amine group formed hydrogen bonds with the main chain of Ser477 and with the side chain of Ser479, as observed during MD simulations (Figure 10C). Although Ser479 is one of the main differences in a sequence compared with GlyT-1, the Ser479Gly mutant displayed only a slightly reduced affinity for ALX-1393 [29,55]. This indicates that the hydrogen bond with this residue is one of the determinants for GlyT-2 selectivity only. The Ser479 side chain creates a steric clash for GlyT-1 inhibitors rather than enhancing the binding of the currently known GlyT-2 inhibitors. The protonated amino group also forms a cation–π interaction with Tyr207; however, it is not very stable. Although not involved in direct interactions, Thr582 confers beneficial hydrophilic/hydrophobic properties on the S1 site for the binding of amino acid fragments of ALX-1393 and its analogs. Replacement of Thr582 with leucine, present in GlyT-1, almost completely inhibited the binding of ALX-1393 to GlyT-2. The aromatic fragments of the described glycine derivatives are located almost entirely in the S2 site. Docking and MD studies of ALX-1393 showed π–π stacking with Tyr286 and Trp482, and additional hydrophobic interactions with Trp215, Tyr287, Phe476, and Leu290 (Figure 9B and Figure 10C). For compound **9**, intensified aromatic and hydrophobic interactions with Trp215, Phe476, and Ile290 were observed (Figure 9D). These derivatives are shorter and more inflexible, contain less extended aromatic fragments compared with GlyT-1 inhibitors, and thus fit better to the reduced S2 site in GlyT-2.

While docking to the inward-open state of GlyT-2, the binding mode for ALX-1393 and its analogs was also fairly consistent (Supplementary Figure S9). The carboxyl group is located at a position corresponding to the sulfonyl group of compound **1** in GlyT-1, and the protonated amino moiety forms hydrogen bonds with the main chain of the nonhelical TM6 fragment. In addition, the arrangement of aromatic rings correlated with the position of aromatic fragments in the crystal structures of GlyT-1. However, the ALX-1393 complex with GlyT-2 in the inward-open state was less stable in MD simulations, and the MM-GBSA energy value was significantly higher compared with that for the complex of this compound with GlyT-2 in the outward-open state (Table 2, Supplementary Figure S10). Thus, the preferred binding site for ALX-1393 and its analogs may be the S1/S2 site, which is consistent with the results of Benito-Munoz et al. [29].

## 3. Methods

### 3.1. Homology Modeling

The amino acid sequences of human GlyT-1 and GlyT-2 transporters were downloaded in FASTA format from the UniProt database. For GlyT-1, we used the sequence of isoform c (GlyT-1c), which occurs in the CNS. Among the crystal structures of the SLC6 family proteins available in the PDB database, four structures of DAT from *Drosophila melanogaster* (PDB codes: 4M48, 4XP4, 4XP9, and 4XPH), one structure of human SERT (PDB code: 5I73), and two structures of human GlyT-1 (PDB codes: 6ZBV and 6ZPL) were retrieved. The amino acid sequences of glycine transporters and templates were aligned using the ClustalW multiple alignment option in BioEdit 7.2.6 program (Supplementary Figure S1). The obtained alignment was verified for the overlapping of conserved residues and compared with other alignments for the SLC6 protein family [53,59,60]. We omitted the *N*- and *C*-terminus due to low sequence homology and a lack of direct engagement in ligand binding. Based on the alignment of sequences, pools of GlyT-1 and GlyT-2 models in the outward-open, partially occluded, and inward-open states were built using the Modeller 9.18 program and the SWISS-MODEL server. Using Modeller, 100 models were generated for each template using MyModel class and the high optimization level. Cysteine residues forming the disulfide bridge within EL2 were defined. Using SWISS-MODEL, one model for each template was obtained. The models preserved sodium and chloride ions found within the binding sites in the templates, whereas ligands were rejected. In the case of models built on the 5I73 template, in which the chloride ion is missing, this ion was transferred to the models from the 4M48 template after superimposing the ion binding sites in the PyMOL program. Based on the DOPE and QMEAN scores, from the 100 models generated in Modeller, one best model was selected for each template for further evaluation with Verify3D program and Ramachandran plots. The models built using the SWISS-MODEL server as well as the templates were assessed in the same manner for comparison of results. Given the relatively good scores, particularly considering the binding sites, the entire pool of 14 models for each type of glycine transporter was submitted to docking studies with an aim of choosing the best models. The assessment of the finally selected models is described in Table S1.

### 3.2. Docking Studies

The ligand 3D structures were created using the Maestro 11.8 program and optimized with the LigPrep module applying the OPLS3e force field. Ionization states were generated at physiological pH (7.4 ± 0.5) using the Epik 4.6 program. The predicted pK_a_ values were verified with the Marvin 21.8 program. For compounds containing chiral atoms with undefined absolute configurations, all possible stereoisomers were generated and used in the docking process.

Models were prepared in the Protein Preparation Wizard using default settings. In all GlyT-1 and GlyT-2 models built on the 4XPH template and GlyT-1 models built on the 5I73 template with SWISS-MODEL server, the side chain of Tyr370 in GlyT-1 or Phe476 in GlyT-2 was in a position that hinders access to the S1 site. Prior to ligand docking, the conformation of these residues was changed in accordance with the crystal structures with an open extracellular gate.

All docking processes were carried out in the Glide 8.1 program. For docking to S1/S2 site, the grid center was defined by the Tyr196 and Tyr370 residues in GlyT-1 and the corresponding Tyr287 and Phe476 residues in GlyT-2. In the case of docking to models in the inward-open state, the grid center was defined by the position of compound **1** transferred from the templates. In all docking processes, the inner box size was 15 × 15 × 15 Å, and the outer box size was 35 × 35 × 35 Å. Ligands were docked using standard precision. Five poses were written out for each ligand. The OPLS3e force field was applied during grid generation and Glide docking. The binding modes were visualized in the PyMOL 2.4.1 program.

### 3.3. Molecular Dynamics

MD simulations were performed in NAMD 2.13 using the CHARMM36m force field. Complexes were positioned in the membrane using the OPM server, and input files for MD simulations were prepared with the CHARMM-GUI online server following the same settings mentioned in our previous papers [53,61,62]. The system was equilibrated via a six-step protocol, and MD simulations were run at 303.15 K with a time step of 2 fs and a total duration of 50 ns. The interval for both the energy and trajectory recordings was 10 ps.

The RMSD for ligands and proteins was analyzed in VMD 1.9.3 after superpositioning all frames on the start frame for the protein backbone. The RMSD for the protein was calculated taking into account both the main and side chains of amino acids located at a distance of 7Å from the initial ligand pose. Hydrogen bonds were mapped in VMD with a defined maximum donor–acceptor distance of 3.5 Å and an angle cutoff of 40°. In the case of ionic interactions, the cutoff for the distance between charged atoms was set to 5 Å. The presence of cation–π interactions was determined based on the distance between the positively charged nitrogen atom and the center of the aromatic ring (<6 Å), whereas aromatic interactions were determined based on the distance between the centers of the two aromatic rings (<5.5 Å). In addition, the relative orientations of the fragments involved in these interactions were visually examined.

The MM-GBSA binding free energy values were evaluated for 21 frames derived from the last 2 ns of the MD simulation (one frame for every 0.1 ns) using the Prime MMGBSA 3.0 program. The VSGB solvation model and OPLS3e force field were applied for this purpose. Protein flexibility, water molecules, and additional sodium and/or chloride ions (apart from the ions initially present at the binding site) were not considered in the calculations. The statistical significance of the difference in MM-GBSA energy values between the compared complexes was verified using a *t*-test in Statistica 13 software.

## 4. Conclusions

Glycine transporters (GlyT-1 and GlyT-2) play a key role in the function of both the inhibitory glycinergic system and the excitatory glutamatergic system. Inhibitors that selectively block these transporters have great potential for application in the treatment of various CNS diseases, such as schizophrenia, drug abuse, epilepsy, and neuropathic pain. Many groups of glycine transporter inhibitors with different selectivity and inhibition kinetics have been discovered so far. However, due to their poor pharmacokinetic and/or pharmacodynamic characteristics, no compound has been approved for therapeutic use to date. Knowledge about the mode of binding of particular inhibitor groups within glycine transporters is still limited, which hinders the search for new compounds with the desired selectivity and properties. The recently released crystal structure of GlyT-1 in the inward-open state revealed an unusual binding mode for a noncompetitive inhibitor within the intracellular release pathway. In this study, we explored in detail the structure of glycine transporters by building homology models of these proteins in different conformational states and investigated their interactions with particular ligands through docking studies and MD simulations.

Our studies indicated that sarcosine-based noncompetitive GlyT-1 inhibitors, such as ORG-24598 and ALX-5407, can bind within the intracellular release pathway of GlyT-1, similar to the analogs of compound **1** from crystal structures. The selective binding of these inhibitors at this site in GlyT-1 can be attributed to a tighter pocket, allowing stronger hydrophobic interactions with the aromatic fragments of ligands, as well as by the presence of a Gly373 residue in place of Ser479 in GlyT-2. Interestingly, ALX-5407 fits equally well at the S1/S2 site of GlyT-1 in the outward-open state, indicating two potential binding sites that may differ in affinity. Competitive inhibitors of GlyT-1 bind coherently within the S1 and S2 sites. In the case of compounds containing a sulfonamide or sulfonyl moiety, the interaction of this fragment mimics that of the substrate carboxyl group by coordinating the sodium ion and forming a hydrogen bond with Gly373. The alkyl or heteroaromatic substituent is positioned in a hydrophobic pocket of the S1 site. Binding of these fragments is hindered by the side chain of Ser479 in GlyT-2 (Gly373 in GlyT-1). Additionally, in GlyT-1, Leu476 allows tighter binding and offers a more hydrophobic environment compared with Thr582 at the corresponding position in GlyT-2. The second region responsible for selectivity between GlyT-1 and GlyT-2, as well as other transporters, is the S2 site within the vestibule. The volume of this site is larger in GlyT-1 due to the presence of Leu524 and Val199 in place of Phe629 and Ile290 in GlyT-2. This enables large aromatic fragments of GlyT-1 inhibitors to bind within it. Competitive GlyT-1 inhibitors containing a protonated amino group form a stable salt bridge with Asp528 from the extracellular gate. ORG-25543, ALX-1393, and their derivatives bind in the outward-open state of GlyT-2. ORG-25543 is mainly located at the S2 site, and its interaction with the carboxyl group of Asp633 via a protonated amino group at this area seems to be crucial. The benzyloxy fragment of this compound is directed toward the S1 site. The selective binding of this fragment to GlyT-2 is likely due to Thr582 which provides more space compared with Leu476 in GlyT-1. ALX-1393 binds more tightly within the S1 site, where its amino acid fragment forms substrate-like interactions, which is reflected by slight differences in inhibition kinetics compared with ORG-255432. In the case of ALX-1393 and its analogs, the shorter linker and less extended aromatic fragments, compared with GlyT-1 inhibitors, allow them to bind within the limited space of the S2 site in GlyT-2.

The glycine transporter models investigated in this study, along with docking studies, MD simulations, and MM-GBSA energy calculations, enabled the determination of the binding modes of many inhibitors. In addition, they allowed us to identify the amino acids that are responsible for the selectivity of glycine transporters and understand the differences in transport kinetics for some groups of inhibitors. The information about the structure of glycine transporters and the algorithm for investigating the binding modes of compounds presented in this paper may be useful in the future to design new GlyT-1 and GlyT-2 inhibitors with desired properties, as well as compounds selective for other transporters from the SLC6 family.

## Figures and Tables

**Table 1:** MM-GBSA binding free energy values for the noncompetitive and competitive inhibitors in a complex with GlyT-1 in the inward-open and outward-open states. MM-GBSA energy value was calculated for the frames derived from the last 2 ns of 50-ns MD simulation.

	Compound	MM-GBSA * dG_bind_ (kcal/mol)		*p* Value **
		Outward-Open	Inward-Open	
non-competitive inhibitors	compound **1**	−54.36 ± 0.42	−73.69 ± 0.80	*p* < 0.0001
	bitopertin	−55.88 ± 0.73	−60.33 ± 1.07	*p* = 0.0014
	ORG-24598	−45.14 ± 1.20	−51.67 ± 0.79	*p* < 0.0001
	ALX-5407	−65.78 ± 1.04	−66.18 ± 0.71	*p* = 0.7519
competitive inhibitors	SSR-504734	−63.93 ± 0.88	−50.80 ± 0.75	*p* < 0.0001
	compound **2**	−74.40 ± 1.19	−60.29 ± 0.84	*p* < 0.0001
	compound **3**	−68.56 ± 0.77	−45.57 ± 0.82	*p* < 0.0001

* mean value ± SEM, ** *t*-test.

**Table 2:** MM-GBSA binding free energy values for ORG-25543 and ALX-1393 in complex with GlyT-2 in the outward-open and inward-open states. MM-GBSA energy value was calculated for the frames derived from the last 2 ns of 50-ns MD.

Compound	MM-GBSA * dG_bind_ (kcal/mol)		*p* Value **
	Outward-Open	Inward-Open	
ORG-25543	−65.90 ± 1.02	−61.31 ± 0.84	*p* = 0.0012
ALX-1393	−68.03 ± 0.94	−39.54 ± 0.96	*p* < 0.0001

* mean value ± SEM, ** *t*-test.

## Data Availability

Data are contained within the article.

## Supplementary Materials

The following supporting information can be downloaded at: https://www.mdpi.com/article/10.3390/ijms23148050/s1.
