# Supplementary material for: Analysis of Binding Determinants for Different Classes of Competitive and Noncompetitive Inhibitors of Glycine Transporters
Source: Int J Mol Sci. 2022 Jul 21;23(14):8050. doi: 10.3390/ijms23148050 (PMC9317360; doi:10.3390/ijms23148050)
Supplement: Supplementary file 1 [file ijms-23-08050-s001.zip › ijms-1808077-supplementary.pdf]

```
hGlyT-1               98   LKRGNWGNQIEFVLTSVGYAVGLGNVWRFPYLCYRNGGGAFMFPYFIMLIFCGIPLFFMELSFGQFASQGCLGVW-RISPMFKGVGYGMM 186
hGlyT-2              189   KARGNWSSKLDFILSMVGYAVGLGNVWRFPYLAFQNGGGAFLIPYLMMLALAGLPIFFLEVSLGQFASQGPVSVW-KAIPALQGCGIAML 277
hGlyT-1 (6ZPL/6ZBV)   98   LKRGNWGNQIEFVLTSVGYAVGLGNVWRFPYLCYRNGGGAFMFPYFIMLIFCGIPAFFMELSFGQFASQGCLGVW-RISPMFKGVGYGMM 186
dDAT (4M48)           25   DERETWSGKVDFLLSVIGFAVDLANVWRFPYLCYKNGGGAFLVPYGIMLAVGGIPLFYMELALGQHNRKGAITCWGRLVPLFKGIGYAVV 114
dDAT (4XP4/4XP9)      25   DERETWSGKVDFLLSVIGFAVDLANVWRFPYLCYKNGGGAFLVPYGIMLAVGGIPLFYMELALGQHNRKGAITCWGRLVPLFKGIGYAVV 114
dDAT (4XPH)           25   DERETWSGKVDFLLSVIGFAVDLANVWRFPYLCYKNGGGAFLVPYGIMLAVGGIPLFYMELALGQHNRKGAITCWGRLVPLFKGIGYAVV 114
hSERT (5I73)          77   GERETWGKKVDFLLSVIGYAVDLGNVWRFPYICAQNGGGAFLLPYTIMAIFGGIPLFYMELALGQYHRNGCISIWRKICPIFKGIGYAIC 166

hGlyT-1              187   VVSTYIGIYYNVVICIAFYYFFSSMTHVLPWAYCNNPWNTHDCAG----VLDASNLTNGSRPAALPSNLSHLLNHSLQRT---------- 262
hGlyT-2              278   IISVLIAIYYNVIICYTLFYLFASFVSVLPWGSCNNPWNTPECKDKTKLLLDSCVISDHPKIQIKNSTFCMTAYPNVTMVNFTSQANKTF 367
hGlyT-1 (6ZPL/6ZBV)  187   VVSTYIGIYYNVVICIAFYYFFSSMTHVLPWAYCNNPWNTHDCAG----VLDASNLT-----------------HSLQRT---------- 262
dDAT (4M48)          115   LIAFYVDFYYNVIIAWSLRFFFASFTNSLPWTSCNNIWNTPNCRP-----FES----------------------------------QGF 208
dDAT (4XP4/4XP9)     115   LIAFYVDFYYNVIIAWSLRFFFASFTNSLPWTSCNNIWNTPNCRP-----FEG--------------------------------HVEGF 208
dDAT (4XPH)          115   LIAFYVGFYYNVIIAWSLRFFFASFTNSLPWTSCNNIWNTPNCRP-----FEG--------------------------------HVEGF 208
hSERT (5I73)         167   IIAFYIASYYNTIMAWALYYLISSFTDQLPWTSCKNSWNTGNCTN-----YFSEDN---------------------------ITWTLHS 224

hGlyT-1              263   -SPSEEYWRLYVLKL--SDDIGNFGEVRLPLLGCLGVSWLVVFLCLIRGVKSSGKVVYFTATFPYVVLTILFVRGVTLEGAFDGIMYYLT 349
hGlyT-2              368   VSGSEEYFKYFVLKI--SAGIEYPGEIRWPLALCLFLAWVIVYASLAKGIKTSGKVVYFTATFPYVVLVILLIRGVTLPGAGAGIWYFIT 455
hGlyT-1 (6ZPL/6ZBV)  263   -SPSEEYWRLYVLKL--SDDIGNFGEVRLPLLGCLGVAWLVVFLCLIRGVKSSGKVVYFTATFPYVVLTILFVRGVTLEGAFDGIMYYLT 349
dDAT (4M48)          209   QSAASEYFNRYILELNRSEGIHDLGAIKWDMALCLLIVYLICYFSLWKGISTSGKVVWFTALFPYAALLILLIRGLTLPGSFLGIQYYLT 298
dDAT (4XP4/4XP9)     209   QSAASEYFNRYILELNRSEGIHDLGAIKWDMALCLLIVYLICYFSLWKGISTSGKVVWFTALFPYAVLLILLIRGLTLPGSFLGIQYYLT 298
dDAT (4XPH)          209   QSAASEYFNRYILELNRSEGIHDLGAIKWDMALCLLIVYLICYFSLWKGISTSGKVVWFTALFPYAVLLILLIRGLTLPGSFLGIQYYLT 298
hSERT (5I73)         225   TSPAEEFYTRHVLQIHRSKGLQDLGGISWQLALCIMLIFTVIYFSIWKGVKTSGKVVWVTATFPYIALSVLLVRGATLPGAWRGVLFYLK 314

hGlyT-1              350   PQWDKILEAKVWGDAASQIFYSLGCAWGGLITMASYNKFHNNCYRDSVIISITNCATSVYAGFVIFSILGFMANHLGVDVSRVADH-GPG 438
hGlyT-2              456   PKWEKLTDATVWKDAATQIFFSLSAAWGGLITLSSYNKFHNNCYRDTLIVTCTNSATSIFAGFVIFSVIGFMANERKVNIENVADQ-GPG 544
hGlyT-1 (6ZPL/6ZBV)  350   PQWDKILEAKVWGDAASQAFYSLGCAWGGLITMASYNKFHNNCYRDSVIISITNCATSVYAGFVIFSILGFMANHLGVDVSRVADH-GPG 438
dDAT (4M48)          299   PNFSAIYKAEVWADAATQVFFSLGPGFGVLLAYASYNKYHNNVYKDALLTSFINSATSFIAGFVIFSVLGYMAHTLGVRIEDVATE-GPG 387
dDAT (4XP4/4XP9)     299   PNFSAIYKAEVWVDAATQVFFSLGPGFGVLLAYASYNKYHNNVYKDALLTSFINSATSFIAGFVIFSVLGYMAHTLGVRIEDVATE-GPG 387
dDAT (4XPH)          299   PNFSAIYKAEVWVDAATQVFFSLGPGFGVLLAYASYNKYHNNVYKDALLTSFINSATSFIAGFVIFSVLGYMAHTLGVRIEDVATE-GPG 387
hSERT (5I73)         315   PNWQKLLETGVWIDAAAQIFFSLGPGFGVLLAFASYNKFNNNCYQDALVTSVVNCMTSFVSGFVIFTVLGYMAEMRNEDVSEVAKDAGPS 404

hGlyT-1              439   LAFVAYPEALTLLPISPLWSLLFFFMLILLGLGTQFCLLETLVTAIVDEVGNEWILQKKTYVTLGVAVAGFLLGIPLTSQAGIYWLLLMD 528
hGlyT-2              545   IAFVVYPEALTRLPLSPFWAIIFFLMLLTLGLDTMFATIETIVTSISDEFP-KYLRTHKPVFTLGCCICFFIMGFPMITQGGIYMFQLVD 633
hGlyT-1 (6ZPL/6ZBV)  439   LAFVAYPEALTLLPISPLWSLLFFFMLILLGLGTQFCLLETLVTAIVDEVGNEWILQKKTYVTLGVAVAGFLLGIPLTSQAGIYWLLLMD 528
dDAT (4M48)          388   LVFVVYPAAIATMPASTFWALIFFMMLATLGLDSSFGGSEAIITALSDEFP-KIKRNR-ELFVAGLFSLYFVVGLASCTQGGFYFFHLLD 475
dDAT (4XP4/4XP9)     388   LVFVVYPAAIATMPASTFWALIFFMMLATLGLDSSFGGSEAIITALSDEFP-KIKRNR-ELFVAGLFSLYFVVGLASCTQGGFYFFHLLD 475
dDAT (4XPH)          388   LVFVVYPAAIATMPASTFWALIFFMMLATLGLDSSFGGMEAIITALSDEFP-KIKRNR-ELFVAGLFSLYFVVGLASCTQGGFYFFHLLD 475
hSERT (5I73)         405   LLFITYAEAIANMPASTFFAIIFFLMLITLGLDSSFAGLEGVITAVLDEFP-HVWAKRRERFVLAVVITCFFGSLVTLTFGGAYVVKLLE 493

hGlyT-1              529   NYAASFSLVVISCIMCVAIMYIYGHRNYFQDIQMMLGFPPPLFFQICWRFVSPAIIFFILVFTVIQYQPITYNHYQYPGWAVAIGFLMAL 618
hGlyT-2              634   TYAASYALVIIAIFELVGISYVYGLQRFCEDIEMMIGFQPNIFWKVCWAFVTPTILTFILCFSFYQWEPMTYGSYRYPNWSMVLGWLMLA 723
hGlyT-1 (6ZPL/6ZBV)  529   NYAASFSLVVISCIMCVAIMYIYGHRNYFQDIQMMLGFPPPLFFQICWRFVSPAIIFFILVFTVIQYQPITYNHYQYPGWAVAIGFLMAL 618
dDAT (4M48)          476   RYAAGYSILVAVFFEAIAVSWIYGTNRFSEDIRDMIGFPPGRYWQVCWRFVAPIFLLFITVYLLIGYEPLTYADYVYPSWANALGWCIAG 565
dDAT (4XP4/4XP9)     476   RYAAGYSILVAVFFEAIAVSWIYGTNRFSEDIRDMIGFPPGRYWQVCWRFVAPIFLLFITVYGLIGYEPLTYADYVYPSWANALGWCIAG 565
dDAT (4XPH)          476   RYAAGYSILVAVFFEAIAVSWIYGTNRFSEDIRDMIGFPPGRYWQVCWRFVAPIFLLFITVYGLIGYEPLTYADYVYPSWANALGWCIAG 565
hSERT (5I73)         494   EYATGPAVLTVALIEAVAVSWFYGITQFCRDVKEMLGFSPGWFWRICWVAISPLFLLFIIASFLMSPPQLRLFQYNYPYWSIILGYAIGT 583

hGlyT-1              619   SSVLCIPLYAMFRLCRTDGDTLLQRLKNATKPSRD 653
hGlyT-2              724   CSVIWIPIMFVIKMHLAPG-RFIERLKLVCSPQPD 757
hGlyT-1 (6ZPL/6ZBV)  619   SSVLCIPLYAMFRLARTDGDTLLQRLKNATKPSRD 653
dDAT (4M48)          566   SSVVMIPAVAIFKLLSTPG-SLRQRFTILTTPWRD 599
dDAT (4XP4/4XP9)     566   SSVVMIPAVAIFKLLSTPG-SLRQRFTILTTPWRD 599
dDAT (4XPH)          566   SSVVMIPAVAIFKLLSTPG-SLRQRFTILTTPWRD 599
hSERT (5I73)         584   SSFICIPTYIAYRLIITPG-TFKERIIKSITPETP 617
```

Figure S1. Amino acids sequence alignment used to build models of glycine transporters.

Table S1. Assessment of the selected models and their templates. Models and matrices were evaluated without considering the *N*- and *C*-terminus and the EL2 loop fragment.

		Verify3D	QMEAN	DopeScore	Ramachandran plot	
					favoured	disallowed
models	GlyT-1 (4M48)	80.96	-3.84	-82413.5	91.5	0.4
	GlyT-1 (6ZPL)	77.65	-4.03	-79792.9	92.3	0.0
	GlyT-2 (4XP9)	88.61	-3.17	-84396.3	93.5	0.0
	GlyT-2 (6ZPL)	81.89	-4.02	-80678.7	93.0	0.0
templates	GlyT-1 (6ZPL)	79.17	-3.83	-79239.2	93.8	0.2
	DAT (4M48)	93.85	-2.68	-83617.4	90.7	0.2
	DAT (4XP9)	91.73	-2.62	-83761.5	94.7	0.0

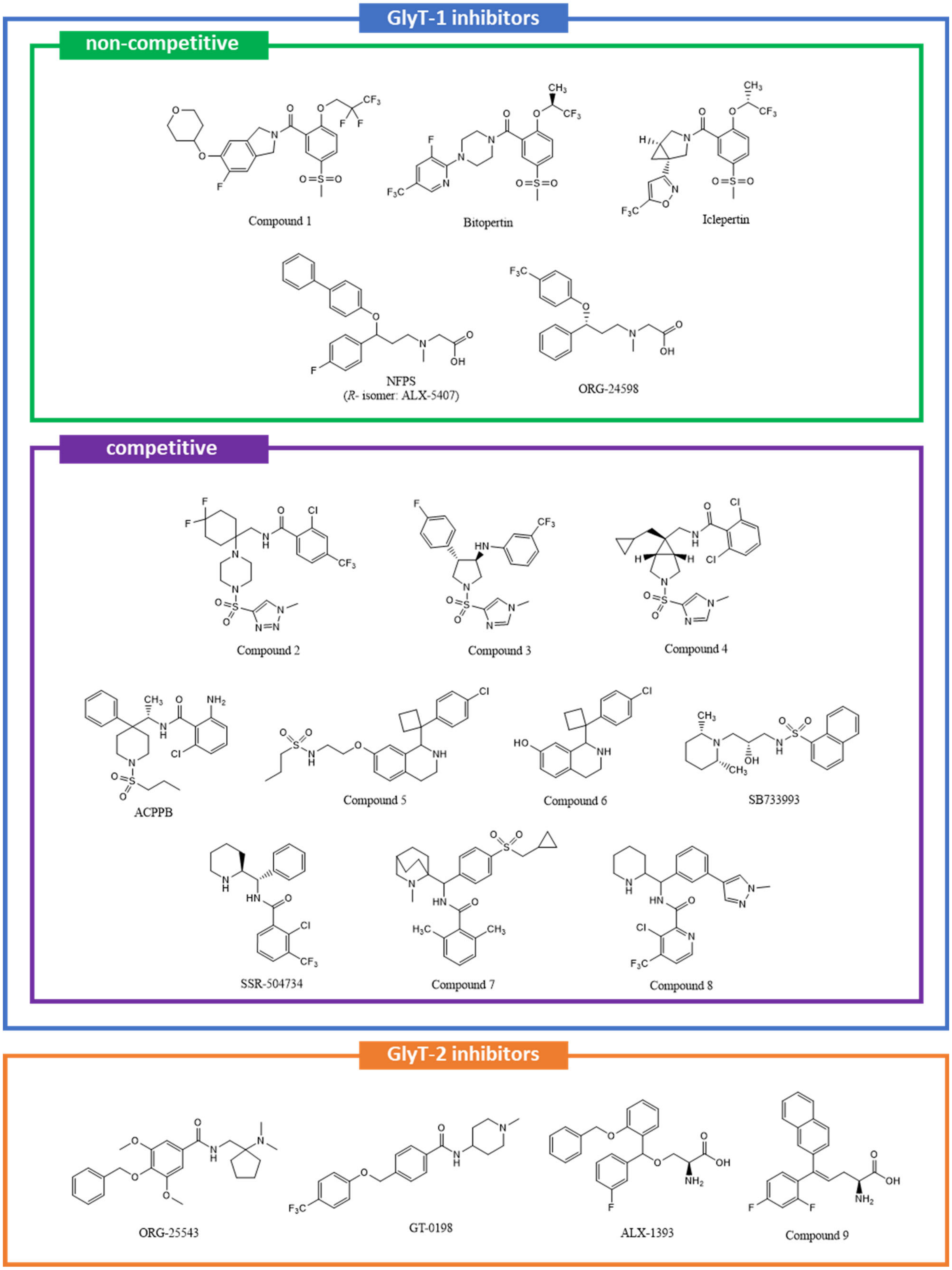

Figure S2. Structures of the compounds for which the detailed binding modes with glycine transporters were presented herein.

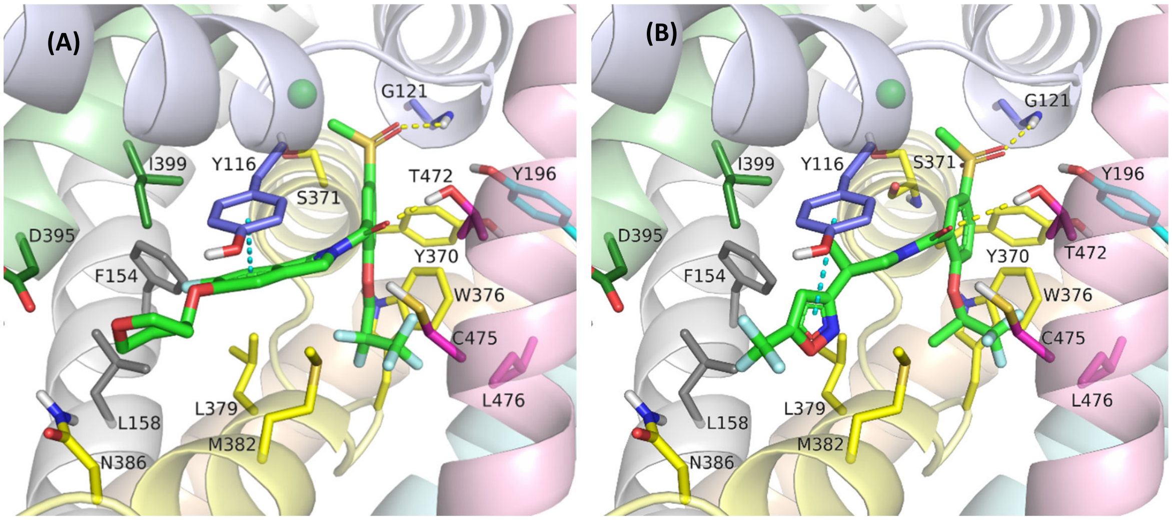

Figure S3. Binding mode of the compound **1** (**A**) and iclepertin (**B**) within GlyT-1 in inward-open state.

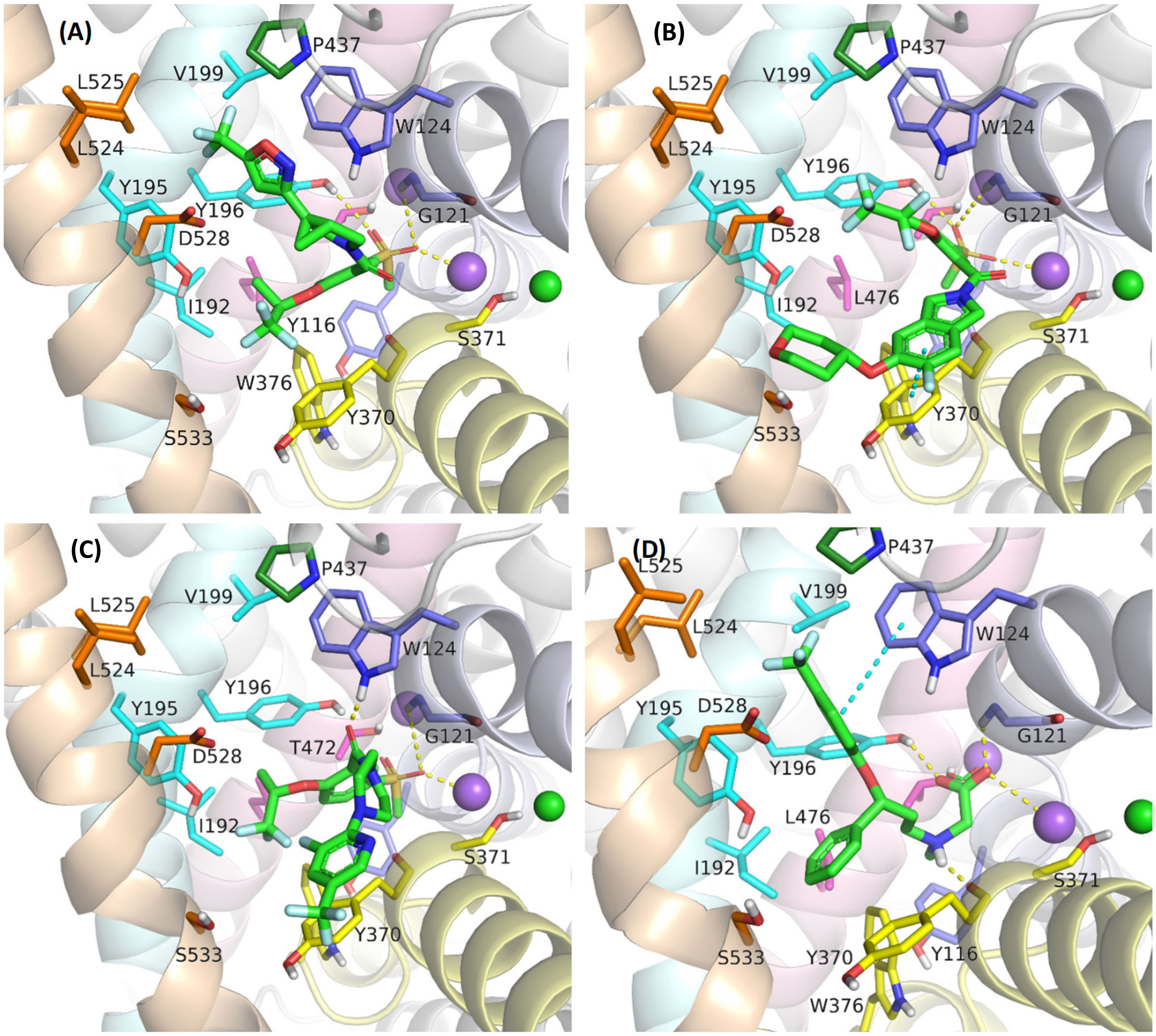

Figure S4. Binding mode of the iclepertin (**A**), compound **1** (**B**), bitopertin (**C**), and ORG-24598 (**D**) within GlyT-1 in outward-open state.

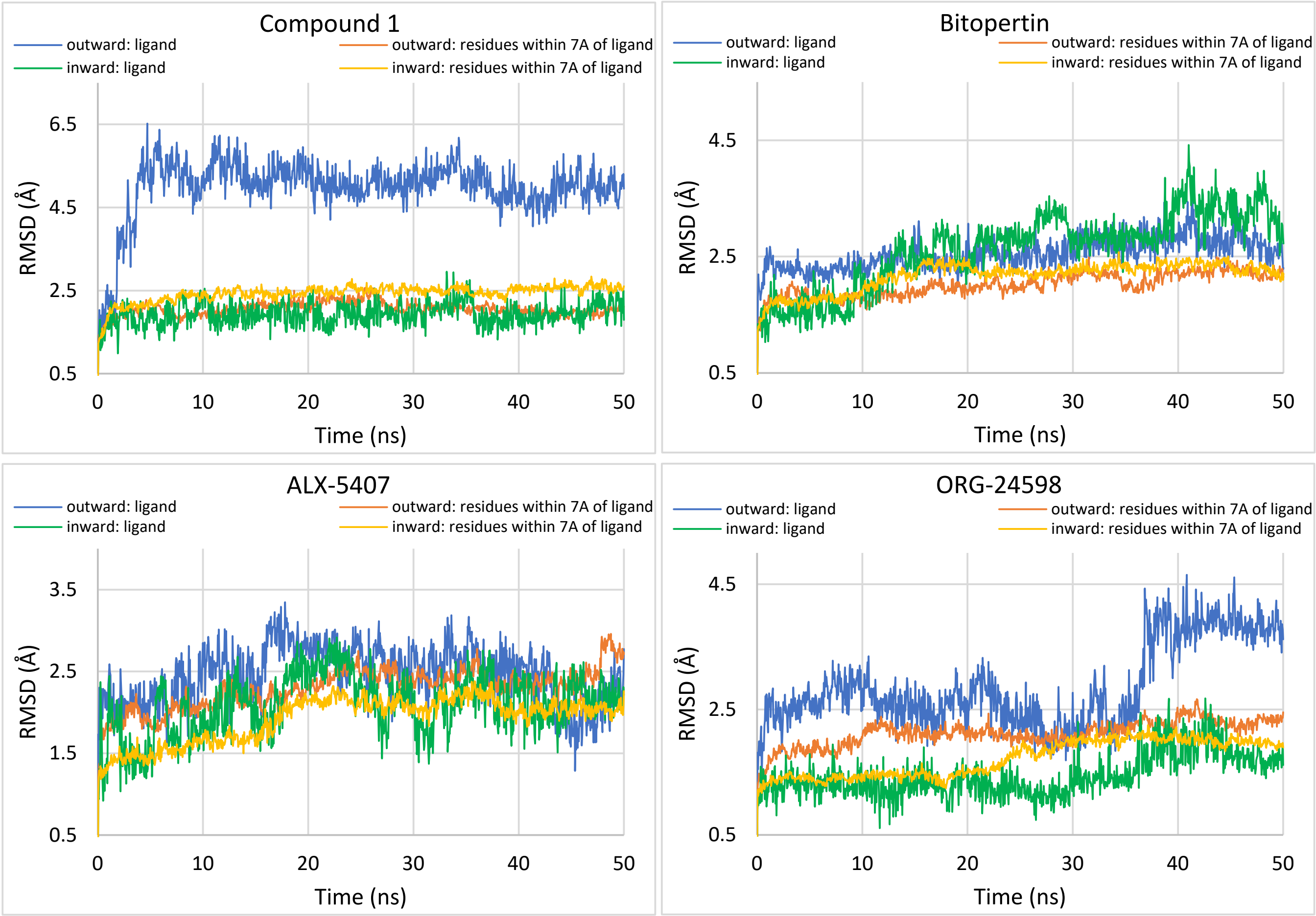

Figure S5. Comparison of RMSD changes in the course of molecular dynamics for compound **1**, bitopertin, ALX-5407, and ORG-24598 in complex with GlyT-1 in outward-open and inward-open states.

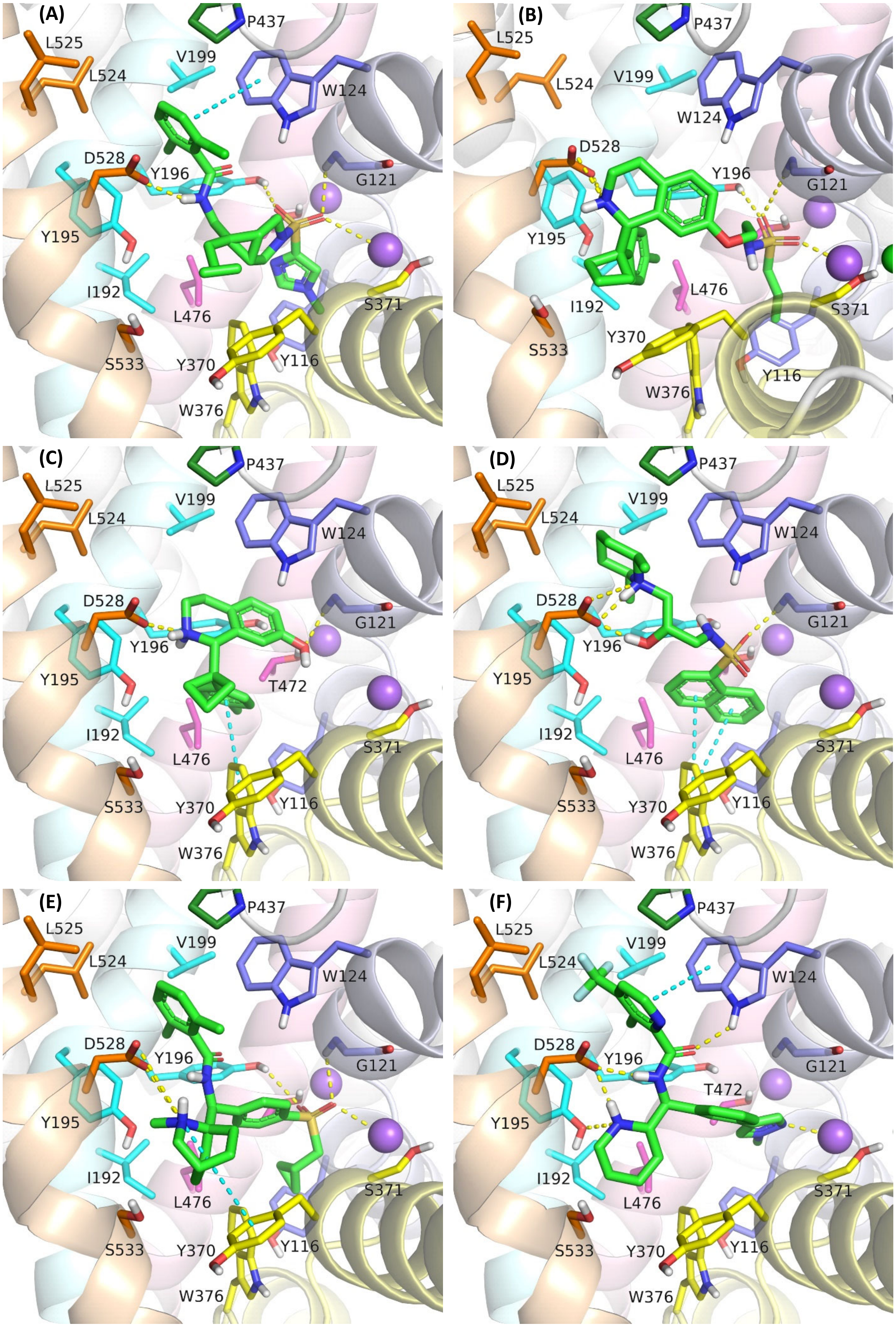

Figure S6. Binding mode of the compound **4** (**A**), compound **5** (**B**), compound **6** (**C**), SB733993 (**D**), compound **7** (**E**), and compound **8** (**F**) within GlyT-1 in outward-open state.

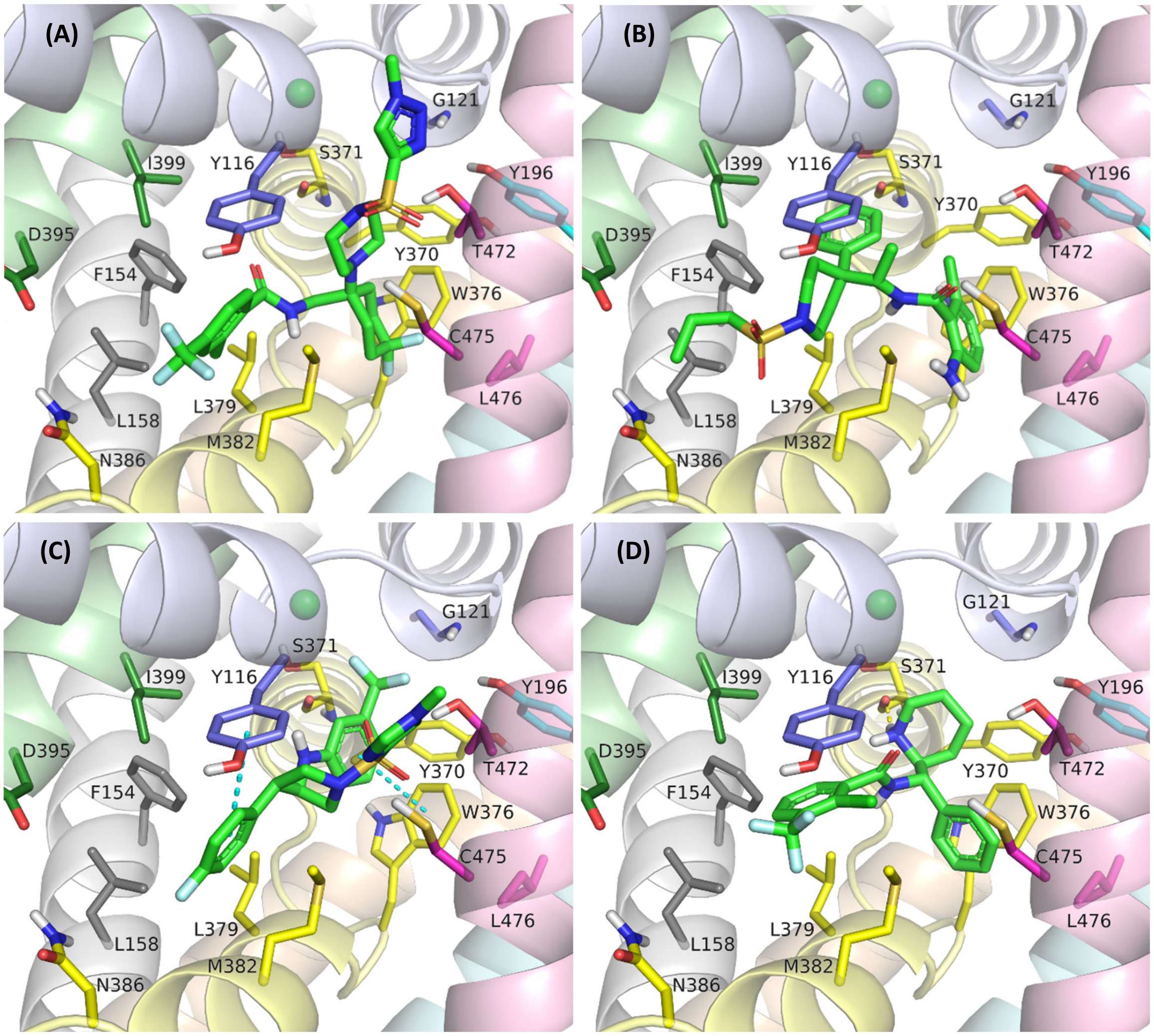

Figure S7. Binding mode of the compound **2** (**A**), ACPPB (**B**), compound **3** (**C**), and SSR-504734 (**D**) within GlyT-1 in inward-open state.

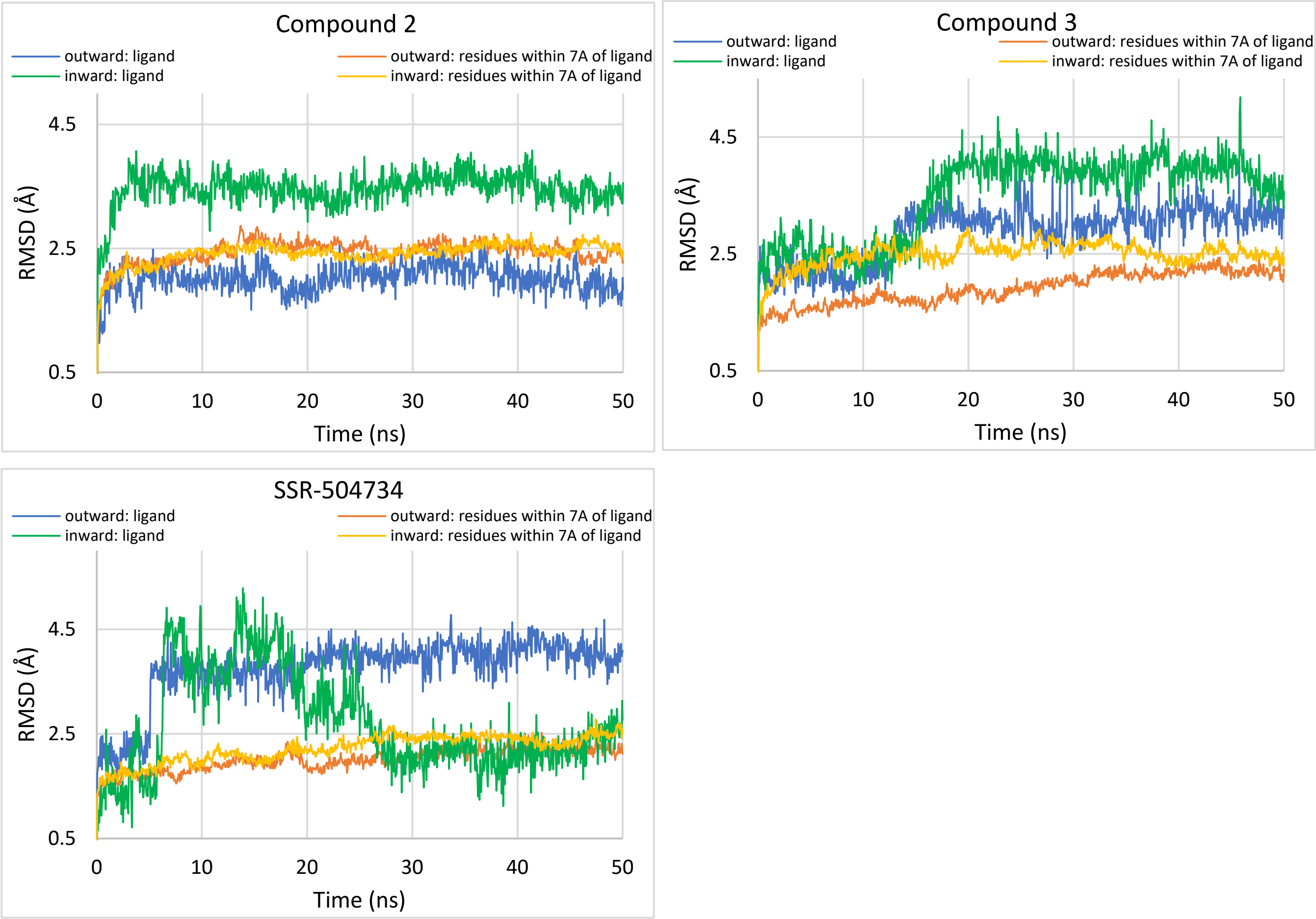

Figure S8. Comparison of RMSD changes in the course of molecular dynamics for compound **2**, compound **3**, and SSR-504734 in complex with GlyT-1 in outward-open and inward-open states.

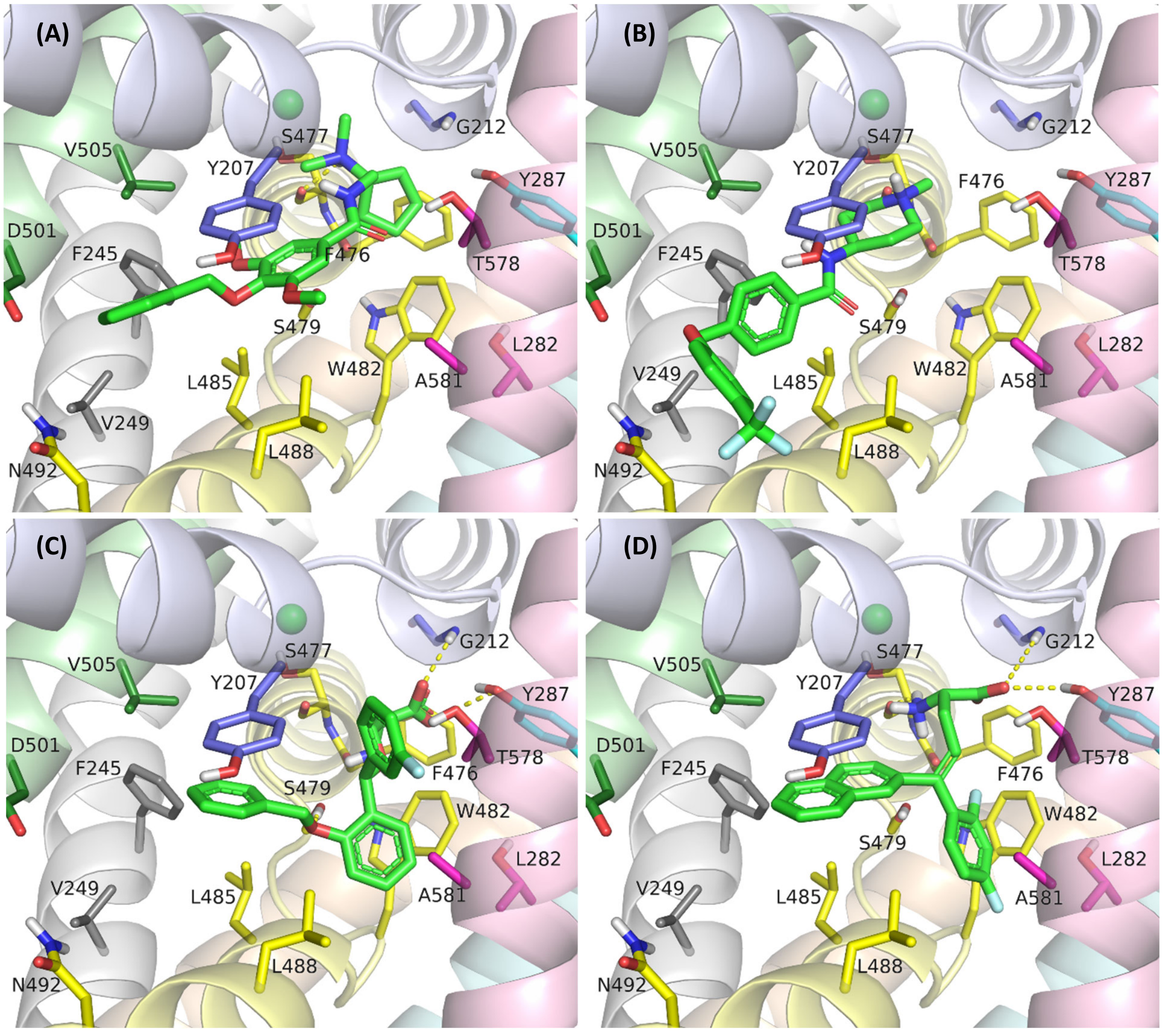

Figure S9. Binding mode of the ORG-25543 (**A**), GT-0198 (**B**), ALX-1393 (**C**), and compound **9** (**D**) within GlyT-2 in inward-open state.

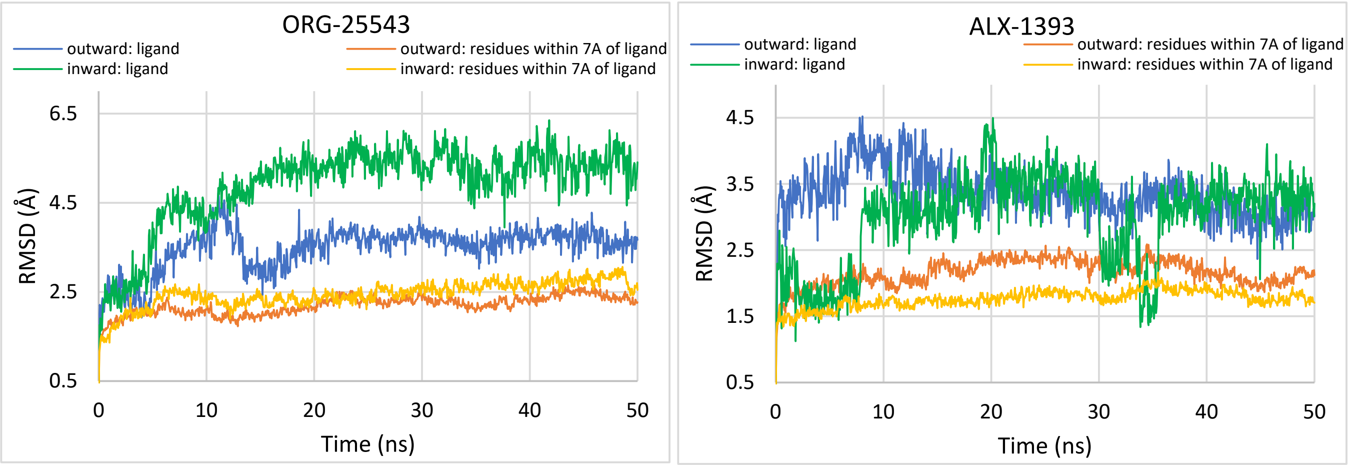

Figure S10. Comparison of RMSD changes in the course of molecular dynamics for ORG-25543 and ALX-1393 in complex with GlyT-2 in outward-open and inward-open states.
